# Supplementary material for: Omega-3 alleviates behavioral and molecular changes in a mouse model of stress-induced juvenile depression
Source: Neurobiol Stress. 2024 May 20;31:100646. doi: 10.1016/j.ynstr.2024.100646 (PMC11190747; doi:10.1016/j.ynstr.2024.100646)
Supplement: Multimedia component 1 [file mmc1.docx]

***Supplementary File***

**Table S1. Sequences of primers for qRT-PCR**

| Gene | Forward primer 5′–3′ | Reverse primer 5′–3′ |
| --- | --- | --- |
| GAPDH | TGCACCACCAACTGCTTAG | GGATGCAGGGATGATGTTC |
| IL-1β | TGTAATGAAAGACGGCACACC | TCTTCTTTGGGTATTGCTTGG |
| TNF | AGCCGATGGGTTGTACCTTG | GTGGGGTGAGGAGCACGTAGTC |
| GSK-3β | TCCATTCCTTTGGAATCTGC | CAATTCAGCCAACACACAGC |

**Table S2 Mean area under receiver operator characteristic (ROC) curve**

|  | **Plasma** | **Liver** | **Brain** |
| --- | --- | --- | --- |
| **Stressed vs.  Not-stressed** | AUC < 0.5NS p = 1 | AUC = 0.56p < 0.001 | AUC = 0.66p < 0.001 |
| **Omega 3 vs.  Vehicle** | AUC = 0.58p < 0.001 | AUC = 0.76p < 0.001 | AUC = 0.76p < 0.001 |
