# Supplementary material for: Omega-3 alleviates behavioral and molecular changes in a mouse model of stress-induced juvenile depression
Source: Neurobiol Stress. 2024 May 20;31:100646. doi: 10.1016/j.ynstr.2024.100646 (PMC11190747; doi:10.1016/j.ynstr.2024.100646)
Supplement: Multimedia component 2 [file mmc2.docx]

**Omega-3 Alleviates Behavioral and Molecular changes in a Mouse Model of Stress-Induced Juvenile Depression**

Tatyana Strekalova^a,b*#^, Daniel Radford-Smith^b*^, Isobel K. Dunstan^b^, Anna Gorlova^c,d^, Evgeniy Svirin^c^, Elisaveta Sheveleva^c,e^, Alisa Burova^c^, Sergey Morozov^c^, [Aleksey Lyundup](https://pubmed.ncbi.nlm.nih.gov/?sort=date&term=Lyundup+A&cauthor_id=38136653)^d,f^, Gregor Berger^g^, Daniel C. Anthony^b#^, Susanne Walitza^g^

^a^Department of Psychiatry and Neuropsychology, Maastricht University, Maastricht, the Netherlands;

^b^Department of Pharmacology, Oxford University, Oxford, UK;

^c^Laboratory of Cognitive Dysfunctions, Institute of General Pathology and Pathophysiology, Moscow, Russia;

^d^RUDN University, 6 Miklukho-Maklaya Str, Moscow, Russia;

^e^Department of Normal Physiology, Sechenov Moscow State Medical University, Moscow, Russia;

^f^Endocrinology Research Centre, Dmitry Ulyanov str. 19,  Moscow, 117036, Russia;

^g^Department of Child and Adolescent Psychiatry and Psychotherapy, University of Zuerich, Zuerich, Switzerland

^*^Equal contribution

**# Joint corresponding authors:**

| Dr. Tatyana Strekalova  Department of Psychiatry and Neuropsychology, Maastricht University, Universiteitssingel, 40  Maastricht, NL 6229 ER the Netherlands  T: +31 43 38 881021  E:[t.strekalova@maastrichtuniversity.nl](mailto:t.strekalova@maastrichtuniversity.nl) | Prof. Daniel C. Anthony  Department of Pharmacology  University of Oxford  Mansfield Road, OX1 3QT  Oxford, UK  T: +44 1865 281136  E: Daniel.anthony@pharm.ox.ac.uk |
| --- | --- |

## Abstract

**Introduction:** Depression is increasingly diagnosed in adolescence, necessitating specific prevention and treatment methods. However, there is a lack of animal models mimicking juvenile depression. This study explores a novel model using ultrasound (US) stress in juvenile mice.

**Methods:** We employed the US stress model in one-month-old C57/BL6 mice, exposing them to alternating ultrasound frequencies (20-25kHz and 25-45kHz) for three weeks. These frequencies correspond to negative and neutral emotional states in rodents and can induce a depressive-like syndrome. Concurrently, mice received either an omega-3 food supplement (FS) containing eicosapentaenoic acid (EPA; 0.55 mg/kg/day) and docosahexaenoic acid (DHA; 0.55 mg/kg/day) or a vehicle. Post-stress, we evaluated anxiety- and depressive-like behaviors, blood corticosterone levels, brain expression of pro-inflammatory cytokines, and conducted metabolome analysis of brain, liver and blood plasma.

**Results:** US-exposed mice treated with vehicle exhibited decreased sucrose preference, a sign of anhedonia, a key feature of depression, increased anxiety-like behavior, elevated corticosterone levels, and enhanced TNF and IL-1β gene expression in the brain. In contrast, US-FS mice did not display these changes. Omega-3 supplementation also reduced anxiety-like behavior in non-stressed mice. Metabolomic analysis revealed US-induced changes in brain energy metabolism, with FS increasing brain sphingomyelin. Liver metabolism was affected by both US and FS, while plasma metabolome changes were exclusive to FS. Brain glucose levels correlated positively with activity in anxiety tests.

**Conclusion:** Chronic omega-3 intake counteracted depressive- and anxiety-like behaviors in a US model of juvenile depression in mice. These effects likely stem from the anti-inflammatory properties of the supplement, suggesting potential therapeutic applications in juvenile depression.

**Keywords:** juvenile depression, Omega-3, eicosapentaenoic and docosahexaenoic acids, ultrasound stress, metabolome, mice

**1.Introduction**

Major Depressive Disorder (MDD), a prevalent mental disorder affecting quality of life, has seen a rise in diagnosis among adolescents over the past decade, as noted by Shorey et al. (2022). This increase has been exacerbated by the COVID-19 pandemic, leading to higher MDD prevalence in youth, as indicated by Oliveira et al. (2022). Prior to the pandemic, 12.9% of European youth exhibited significant depressive symptoms (Lu, 2019); this figure climbed to 20.5% in 2021 (Racine et al., 2021) and further to 27% in 2023 (Maggu et al., 2023). Research has shown that the youth are particularly vulnerable to MDD and anxiety disorders during the pandemic, with an increased risk of suicide (Madigan et al., 2023; Witteveen et al., 2023; Souza et al., 2023). Thus, the rising incidence of MDD in adolescents is a pressing social and medical issue, affecting individuals, their communities, and society at large.

Despite the availability of various depression therapies for adults, many are ineffective compared to vehicle and are not approved for adolescents (Janiaud et al., 2017; Locher et al., 2017). Adolescents with MDD generally show poorer responses to standard antidepressant therapies than adults (Kronenberg et al., 2007; Hankin et al., 2015; Janiaud et al., 2017; Locher et al., 2017). Notably, a meta-analysis by Cipriani et al. (2016) found that only fluoxetine significantly outperformed vehicle. Currently, fluoxetine (in the USA) and escitalopram (in the EU) are the recommended antidepressants for juvenile depression. Adolescent patients typically exhibit more physical disturbances (such as changes in energy, weight, appetite, and sleep) and less frequently show motivational and cognitive issues compared to adults (Rice et al., 2019; Zwolinska et al., 2023). The efficacy and tolerability of antidepressants in young people are debated, given the high vehicle response rate and the associated suicide risk with standard MDD pharmacotherapy (Cipriani et al., 2016; Masi, 2022; Meister et al., 2020; Sher, 2011; Spielmans et al., 2020).

The use of classic antidepressants, particularly SSRIs, in treating adolescent depression is controversial due to the increased suicide risk (Bridge et al., 2007; Li et al., 2022). Additionally, chronic antidepressant treatment in adolescents can interfere with physiological growth and development, potentially causing somatic problems such as altered insulin resistance, metabolic issues, and developmental concerns (Baune et al., 2012; WHO, 2012, 2017; Zuzarte et al., 2018).

Omega-3 fatty acid supplementation as an adjunct therapy for depression is well-established (Chang and Su, 2020). Literature reviews suggest significant therapeutic effects of omega-3-rich supplements, especially when combined with standard antidepressants, in treating depressed children and preadolescents (Pruneti and Guidotti, 2023). Both EPA and DHA have shown potential in improving symptoms in young MDD patients, particularly those with high inflammation or a low baseline Omega-3 index (Chang and Su, 2020). Their distinct anti-inflammatory and immunomodulatory properties raise questions about the optimal ratio for treatment. Currently, a combination of EPA and DHA (1,000-2,000 mg/day, with a 2:1 ratio) is recommended for adolescent MDD patients for 12-16 weeks (Chang and Su, 2020).

The existing literature concerning the use of omega-3 in people includes seven randomized controlled trials (Thakur et al., 2023), but there was significant heterogeneity in the trial designs. For example the trials have employed varying diagnostic criteria for depression (and assessment scales), different age ranges, participants have come from diverse backgrounds. Moreover, there has been no standard for omega-3 dosage or the controls employed. In the largest and most recent study to be performed to date, on 233 young people, no effect of a 12-week-long course of supplementation was measurable compared to placebo (Amminger et al., 2024). However, the baseline levels of omega-3 fatty acids in the treated MDD patients were well within the normal range, which might explain the lack of any significant outcome in this study. Other clinical studies, in which the levels of total n-3 PUFAs, DHA, and EPA were lower in patients with MDD, showed beneficial effects of omega-3 treatment (Lin et al., 2010). Thus, omega-3 supplementation remains of interest as alternative/adjunct medication for depressed patients. Further clinical trials that are ongoing (Häberling et al., 2019). However, the molecular mechanisms that are proposed to underpin the effects of omega-3 in MDD are still unclear.

Animal models, particularly those using stress paradigms, are crucial in understanding the mechanisms of adolescent depression and of the mode of action of new therapies, such as omega-3. Recent studies have highlighted the strong link between adolescent MDD and emotional stress, such as sexual abuse, family member death, and emotional abuse (LeMoult et al., 2020). Animal model of MDD often use stress, a significant factor in MDD etiology, particularly in younger individuals (Hankin et al., 2015; Yang et al., 2015; LeMoult et al., 2020). However, replicating ‘emotional stress’ in rodents has been challenging. Current rodent models have primarily employed physical stressors and, thus, have limited relevance in simulating juvenile MDD.

Recently, animal models based on naturalistic stressors mimicking ‘emotional stress’ have gained more acceptance. For example, the study of Nakatake et al. (2020) showed that only social defeat stress, as a model of emotional stress, led to anhedonia in juvenile mice, compared to the use of physical stressors, which did not. The ultrasound stress (US) paradigm is an example of an ‘emotional stress’ paradigm in which mice are exposed to negative and neutral emotional ultrasound signals over 21 days. The model was devised based on the observation that mice respond differently to sounds within the ultrasound frequency ranges. For example, mice emit sounds in the range of 20–25 kHz in life-threatening situations, such as maternal separation, predation, social defeat, or pain (reviewed in Gorlova et al., 2023). Our previous studies have shown that adult mice exposed to this frequency range (20–25 kHz) exhibited depressive-like and anxiety-like behaviors, in addition to physiological, hormonal and molecular changes, including neuroinflammation and oxidative stress (Strekalova et al., 2018; Gorlova et al., 2019, 2023; Pavlov et al., 2019, 2023, Sambon et al., 2020; de Munter et al., 2021). More specifically, exposure to artificially generated ultrasound (20–25 kHz) in male mice was associated with upregulated corticosterone levels (Pavlov et al., 2019), activated hippocampal microglia, upregulated IL-1β and IL-6 production locally in the hippocampus and within the systemic circulation (Costa-Nunes et al., 2020; de Munter et al., 2021; Pavlov et al., 2023), and increased content of protein carbonyl in limbic structures, which is a marker of oxidative stress (Gorlova et al., 2019; Sambon et al., 2020). Notably, ultrasound (US)-exposed male mice developed prominent depressive-like and anxiety-like behavioral changes (Strekalova et al., 2018; Sambon et al., 2020; de Munter et al., 2021) that were counteracted by long-term administration of the classic antidepressant fluoxetine (Morozova et al., 2016) and by treatments with antioxidants (Sambon et al., 2020; Costa-Nunes et al., 2020; de Munter et al., 2021).Thus, US provides a closer analogy to human emotional stressors and recapitulates the key features of MDD and their pharmacological sensitivity in adult rodents.

## In this study, we sought to investigate whether chronic ultrasound exposure affects adolescent mice in a similar way to adult mice. Given the ongoing development of the mouse nervous systems that is characterised by the refinement of cognitive circuitry and increased myelination (Hueston et al., 2017 Chahboune et al., 2007; Semple et al., 2013), we hypothesized that such an environment can render the adolescent brain potentially more vulnerable to ultrasound stressors. We anticipated that as for adult mice, juvenile mice subject to unpredictable ultrasound between 20 and 25 kHz would display depressive-like changes at the behavioral, molecular, and metabolic level. We also addressed the question as to whether the extended administration of omega-3 containing PUFA might counteract these changes.

**2.Theory/calculation**

## Given the ongoing development of the mouse nervous systems, we hypothesized that the impact of ultrasound stressors might be particularly significant during this stage. Thus, in this study, we sought to investigate whether chronic ultrasound exposure affects adolescent mice similarly to adults and whether omega-3 food supplementation can mitigate the MDD-like changes in the mouse US-stress-induced model of depression. We report the effect of omega-3 and US stress on the expression of pro-inflammatory cytokines, blood corticosterone levels, and the NMR-based metabolome of the brain, liver, and blood.

## 3. Materials and Methods

### 3.1 Experimental animals and conditions

For stress experiment, 40 1-month-old C57BL/6 male mice were obtained from Charles River certified supplier (http://www.spf-animals.ru/about/providers/animals). Mice were single housed with to ensure controlled exposure to the ultrasound stress and free access to the diets, under standard laboratory conditions as described elsewhere with food and water provided ad libitum;

light on at 9:00, light out at 19:00. All protocols complied with Directive 2010/63/EU of 22 September 2010, 2010/63/EU and ARRIVE guidelines (<http://www.nc3rs.org.uk/arrive-guidelines>). Experiments were carried out under the approval of the local veterinarian committee of MSMU (22/10/17‐MSMU‐35). All efforts were undertaken to minimize the potential discomfort of animals during the study.

### 3.2 Study design

Four groups of mice that were balanced upon body weight, of ten animals each, were formed: control (non-stressed) vehicle-treated, control (non-stressed) FS-treated mice, US (stressed) vehicle-treated, US (stressed) FS-treated (Fig. 1A). US-groups of mice were subjected to a 21-day exposure of ultrasound of unpredictably alternating frequencies as described elsewhere (Gorlova et al., 2019; Pavlov et al., 2023; see below). Concomitantly with US-exposure, mice assigned to FS-treated groups were housed on a custom-made diet containing FS, or on custom-made diet containing vehicle. Simultaneously with their exposure to ultrasound, mice assigned to the treatment groups were fed the FS diet that included eicosapentaenoic acid (EPA; 0.55 mg/kg/day) and docosahexaenoic acid (DHA; 0.55 mg/kg/day), standard laboratory chow, or a custom-made diet labelled as 'vehicle' (Burgerstein GmbH, Vienna Austria). The vehicle diet consisted of medium-chain triglycerides (MCT) without any unsaturated fatty acids, and a small quantity of fish oil was added to replicate the flavour of the FS diet. To obtain custom-made diet pellets, the content of respective oil-based 5 g capsules containing FS or vehicle (Burgerstein GmbH, Vienna, Austria) was mixed with a standard laboratory chow and diet grains were produced as described elsewhere (Costa-Nunes et al., 2014). The dosage of FS was calculated according to FDA practice with rodent translational studies, and was based on 13-fold dosage increase for murine application vs. recommended human dose. Body weight and diet intake were monitored weekly though out the study. After the termination of US, all mice were studied in a sucrose test for hedonic state (day 22), Novel cage explorative activity test (day 23), Dark‐light box anxiety test (day 24), Open field (day 25). All behavioral experiments were carried out during the dark phase of light/dark cycle. On day 26, all animals were killed, their blood was collected for a metabolome study and cortisol (CORT) ELISA determination. Brains were dissected and prefrontal cortex (PFC) and the hippocampus were isolated for RT-qPCR and metabolome studies. Liver was dissected for metabolome assay as well.

### 3.3 Ultrasound stress

US procedure was carried out as described elsewhere (Strekalova et al., 2018; Pavlov et al., 2019, 2023). For a 21-day period, ultrasound radiation of average intensity of 50±5 dB and variable frequencies in a 20–45 Hz range was constantly delivered within a laboratory environment to experimental groups of mice using a random schedule of alternating frequencies via a commercially available device (Weitech, Wavre, Belgium). The range of ultrasound stimulation frequency was alternated every 10 min between frequencies 20–25kHz, 25–40kHz and 40–45 kHz. The shape of the ultrasound signal was fluctuating, thus, mimicking natural ultrasonic vocalizations of mice (Constantini & D’Amato, 2006). The selectivity of the adverse effects of low-frequency ultrasound during the radiation period versus the potential general negative effects of a constant white noise accompanying the procedure described here was demonstrated previously (Morozova et al., 2016).

### 3.4 Sucrose test

During sucrose test, mice were given, for 8 h, a free choice between two bottles, one with 1% sucrose solution and another with tap water. To prevent possible effects of side preference in drinking behavior, the position of the bottles was switched after 4 h. No previous food or water deprivation was applied before the test; other details of the protocol were used as described elsewhere (Strekalova et al., 2010, 2022). The consumption of water and sucrose solution was estimated simultaneously in control and experimental groups by weighing the bottles. The preference for sucrose was calculated as a percentage of consumed sucrose solution of the total amount of liquid drunk. According to the results of the test, animals were stratified to ‘anhedonic’ and ‘resilient’, taking the minimal value of a sucrose preference in control vehicle-treated mice as a criterion of occurrence of hedonic deficit, as described elsewhere (Strekalova et al., 2004, 2011, 2022) that in the current study was ≤60%.

### 3.5 Novel cage test

In this test, a mouse was placed in a clear plastic cage (14x21x27 cm) with a small amount of fresh litter. For 5 minutes, the number of rears was counted under red light as described elsewhere (Strekalova and Steinbusch, 2010).

### 3.6 Dark‐light box

The apparatus consisted of a dark chamber and an illuminated chamber (600 lux). Mice were introduced to the dark compartment and were allowed to move freely between the two chambers. Latency to exit to the lit compartment, time spent therein and the number of exits to the lit box were recorded for 5 minutes as described elsewhere (Strekalova and Steinbusch, 2010).

### 3.7 Open field test

The open field test was carried out in square box (45x45x45cm) that was illuminated with white light of subtle intensity (5 lux), as described elsewhere (Strekalova et al., 2005). Each animal was placed near the wall and its behavior was recorded a 5 min period. Using previously validated methods with Ethovision program (Ethovision Program 6.95, Noldus, Wageningen, the Netherlands) number of crossed sectors (2 x 2 cm each), time spent in the central area of the arena (15 x 15 cm), the duration of freezing and grooming behaviors and the number of rears were scored as described elsewhere (Malatynska et al., 2012; Strekalova et a., 2021).

### 3.8 Killing of mice and tissue and blood collection

Mice were terminally anesthetized by isoflurane as described elsewhere (Gorlova et al., 2019). Blood collection was performed transcardially, blood was stored in heparinized vials prior to centrifugation (1500 rcf, 15 min, 4°C); plasma was removed and immediately stored at −20°C until use. Thereafter, mice were perfused with NaCl, brain and liver were removed, hippocampus and PFC were dissected and frozen immediately of dry ice and stored at -80C until use.

### 3.9 ELISA CORT assay

To study the concentration of cortisol in blood plasma, mouse enzyme-linked immunosorbent assay (ELISA) was performed using Invitrogen™ Cortisol Competitive ELISA Kit (Thermo Fisher Scientific, MA, USA) according to the manufacturer's instructions. The microwell absorbance was measured at 450 nm with Synergy 4 Hybrid Multi-Mode Microplate Reader (BioTek, Thermo Fisher Scientific, MA, USA) as described elsewhere (Couch et al., 2013).

### 3.10 Quantitative real-time PCR

Total mRNA was isolated from each sample with RNeasy Lipid Tissue Mini Kit (Qiagen, Hilden, Germany). During first-strand cDNA synthesis 1 μg total RNA was converted into cDNA using QuantiTect Reverse Transcription Kit (Qiagen, Hilden, Germany). qRT-PCR was performed using the SYBR Green master mix (Bio-Rad Laboratories, Philadelphia, PA, USA) and the ProFlex PCR system (Thermo Fisher Scientific, MA, USA). qRT-PCR was performed in a 10 μl reaction volume containing a SYBR Green master mix (5 μl), RNase-free water (3 μl), specific forward and reverse primers used at the concentration 20 pmol/μl (1 μl), cDNA (1 μl). Glyceraldehyde-3-phosphate dehydrogenase (GAPDH) was selected as a reference gene, since in previous experiments it was observed relatively low variability in its brain expression (Gorlova et al., 2019). The initial denaturation step for qRT-PCR was at 95°C for 4 min followed by 40 cycles of denaturation at 95°C for 20 seconds, annealing was at 54°C for 90 seconds. Sequences of all primers used are listed in Table S1 (see Supplementary file). All samples were run in triplicate. Data were normalized to GAPDH mRNA expression and calculated as relative-fold changes compared to control vehicle-treated mice, as described elsewhere (Gorlova et al., 2019; Pavlov et al., 2019, 2023).

### 3.11 High-field NMR spectroscopy

Approximately 68mg (SD 16.7mg) fresh snap-frozen liver or brain homogenate (containing PFC and hippocampus) was homogenized with a pestle and mortar on dry ice. Tissue was then diluted 10uL/mg in 1:1 acetonitrile:ddH_2_O and vortexed. Samples were centrifuged for 5 minutes at 5,000 x g at 4°C. 750µL of supernatant was then collected, snap frozen, lyophilized, and stored at -80°C until the day of NMR analysis. Lyophilized samples were then reconstituted in 600μL of 75mM 5:1 disodium phosphate [Na_2_HPO_4_] and monosodium phosphate [NaH_2_PO_4_] in 100% D_2_O, pH = 7.4. Lastly, samples were centrifuged at 2,500 x g for 5 minutes at 4°C before transferal to a 5mm NMR tube (Merck) using a glass pipette dropper. For analysis of plasma, 100µL plasma was added to 500µL 75mM NMR buffer. This mixture was transferred to a 5mm NMR tube (Merck).

^1^H NMR spectra were acquired using a 700 MHz Bruker AVII spectrometer operating at 16.4 Tesla equipped with a ^1^H (13C/15N) TCI cryoprobe, as previously described (Chan et al., 2020; Probert et al., 2022). Sample temperature was stable at 310K. ^1^H NMR spectra were acquired by using a one-dimensional nuclear Overhauser effect spectroscopy (NOESY) presaturation scheme for attenuation of the water resonance with a 2 second presaturation. For liver samples, an addition pulse sequence, Painless-II was applied with 32 scans, an acquisition time of 1.5s, a relaxation delay of 2s, and an inter-pulse delay of 287μs. For plasma, a spin-echo Carr-Purcell-Meiboom-Gill (CPMG) sequence was used under the same conditions as Wasted-II, but with a longer pulse interval of 400μs.

Acquired spectra were phased, baseline corrected, and chemical shifts referenced to the lactate-CH_3_ doublet resonance at δ = 1.33 ppm in Topspin 4.0 (Bruker, Germany). Spectra were then exported to ACD/Labs Spectrus Processor Academic Edition 12.01 (Advanced Chemistry Development, Inc.) and subjected to manual bucketing of each resonance signal (excluding the water region). The integral of each bin was normalised to the sum of all integrals in the spectrum of each sample. Resonances were assigned by referring to the Human Metabolome Database (Wishart et al., 2018), reference to literature values (Govindaraju et al., 2000; Soininen et al., 2009; de Graaf et al., 2014; Cui et al., 2020), spiking of known compounds, and inspection of 2D TOCSY spectra.

### 3.12 Statistical analysis

Behavioral, RT-qPCR, ELISA, and univariate metabolite data were analyzed with GraphPad Prism 6.00 software (San Diego, USA) with two-way ANOVA. Post-hoc Tukey’s multiple comparisons test was used in case of significant factor interaction; Šídák's multiple comparisons test was applied for cases with only one significant factor. All results are presented as Mean±SEM. Spearman’s rank correlation coefficient was used to determine the correlation between behavioral outcomes and brain, liver, and plasma metabolites. The significance level was set at p < 0.05.

Metabolomic data were exported to Rv4.1.3 for statistical analysis. Initially, principal component analysis (PCA) was performed using the “ropls” package to visualise the data in an unsupervised manner. Subsequently, random forest methods (using the “random forest” package) were used to generate algorithms classifying stressed mice against non-stressed mice, as well as mice treated with Omega 3 against mice treated with vehicle only. To build each random forest model, mtry was set to the square-root of the number of predictor variables inputted into the model. The number of trees was fixed in all models at 500.

For each random forest analysis, data from either liver, plasma, or brain were subjected to a 4-fold cross-validation procedure. This cross-validation was repeated 200 times, resulting in an ensemble of 1000 models. The mean accuracy, sensitivity, specificity, and area under the receiver operator characteristic curve (ROC AUC) were reported alongside the most important variables as determined by the mean decrease in Gini coefficient across the 1000 model ensemble. Lastly, to ensure that the random forest models were not overfitting and producing an inflated accuracy, a model with randomly permuted classes was fitted and tested in parallel.

## 4. Results

4.1 Sucrose preference test

Two-way ANOVA demonstrated significant stress effect and stress x treatment interaction, as well as significant treatment effect (F=5.325, p=0.0272, F=4.25, p=0.047 and F=4.190, p=0.0489, respectively) on sucrose preference, which was significantly decreased in the US vehicle-treated group compared to both control vehicle-treated group (p=0.0198, post hoc Tukey’s test) and US-exposed FS-treated group (p=0.037; Fig.1B,D). Mice were assigned to ‘anhedonic’ and ‘resilient’ subgroups using a 60% criterion of anhedonia, corresponding to the lowest value of sucrose preference in control vehicle-treated group (Fig. 1 B). In stressed groups, percent of mice classified as anhedonic was significantly lower in FS group than in vehicle-treated group (p=0.03, Fisher’s exact test; Fig.1C).

No significant treatment effect and stress x treatment interaction were revealed in sucrose intake (F=1.568, p=0.2191 and F=1.269, p=0.2679, respectively, two-way ANOVA), while a significant effect of stress was found (F=4.800, p=0.0356; Fig. 1E). There was no significant effect of treatment on this measure (F=2.068, p=0.1455; Fig. 1E). For water intake, significant stress effect and stress x treatment interaction was present (F=4.236, p=0.0476 and F=4.565, p=0.0401, respectively), a trend for treatment effect was also demonstrated (F=2.773, p=0.1053). This measure was significantly higher in US-stressed vehicle-treated group than in respective control (p=0.0299, post hoc Tukey’s test; Fig. 1F). No significant stress effect, treatment effect or their interaction were shown for total intake (F=0.0186, p=0.8924; F=0.08393, p=0.7739 and F=0.8626, p=0.3598, respectively, two-way ANOVA; Fig. 1G). No other significant changes were present in the sucrose test.

### 4.2 Changes in emotionality and locomotion

4.2.1 Dark-light box

For the latency to exit recorded in this test, two-way ANOVA revealed a significant effect of stress, but no significant effect of treatment or a stress x treatment interaction effect on the latency to enter the lit compartment (F=5.500, p=0.0250, F=0.0002378, p=0.9878, F=0.005872, p=0.9808, respectively; Fig.2A). There was significant effect of the treatment on the time spent in the lit box (F=4.564, p=0.0395, two-way ANOVA), and a trend suggestive of an interaction of the two factors (F=2.761, p=0.1053, F=2.796, p=0.1032, respectively; Fig.2B). Two-way ANOVA did not show significant effect of stress, treatment or an interaction in the number of exits to the light box (F=0.1108, p=0.7412, F=0.03745, p=0.8477, F=0.01534, p=0.9021, respectively; Fig.2C). No other significant group differences were found in the dark-light test.

4.2.2 Open field test

Two-way ANOVA revealed a significant effect of treatment on the number of sectors crossed (F=4.305, p=0.0452) and strong trends toward an effect of stress and stress x treatment interaction (F=3.314, p=0.0770, F=3.3194, p=0.0823, respectively). There was a significant difference between the US-vehicle and US-FS groups, and control-FS and US-FS groups (p=0.0458 and p=0.0434, respectively, Tukey’s test; Fig.3D). We found significant effects of treatment and stress on the time spent in the center of the open field, but there was no significant interaction between these measures (F=9.625, p=0.0037, F=10.39, p=0.0027, F=0.8653, p=0.3585 respectively Fig.2D). Post-hoc testing demonstrated a significantly shorter time was spent in the open field centre in the US-vehicle-treated group than in the control vehicle-treated group (p=0.0281) and in US-FS-treated animals (p=0.0345), while no such differences were found between the two latter groups (p=0.9988). There was also significant difference between US-vehicle-treated animals and control-FS-treated mice (p=0.0004) for lines crossed.

The duration of freezing was significantly affected by treatment and stress (F=6.159, p=0.0179 and F=26.85, p<0.0001, respectively; Fig.2E), but there was no significant interaction (F=37.16, p=0.5460). The US-vehicle-treated group exhibited more freezing behavior than the control vehicle-treated group (p=0.0013, Tukey’s test), but US-FS-animals did not significantly differ from the control vehicle-treated group (p=0.1465). The duration of freezing was significantly shorter in control-FS group than in the US-vehicle-treated group (p<0.0001) and in the US-FS group (0.0134).

Two-way ANOVA revealed significant effects of treatment, stress and an interaction on the frequency of grooming events (F=5.591, p=0.0446, F=9.057, p=0.0119, F=38.86, p<0.0001, respectively Fig.2F). Grooming events were significantly reduced in the US-vehicle-treated group than in the control vehicle-treated mice (p= p<0.0001 Tukey’s test), control vehicle-treated animals (p=0.01), and the US-FS mice (p= p<0.0001). There was a significant effect of stress on the number of rears (F=5.329, p=0.0268), but the effects of treatment and stress x treatment interaction were not significant (F=0.9920, p=0.3259, F=0.3033, p=0.5852, respectively Fig.2G). No other significant group differences were shown for any other recorded parameters in the open field.

### 4.2.3 Novel cage test

### In the novel cage test, no significant effects of treatment, stress or an interaction were revealed by two-way ANOVA (F=0.02124, p=0.8849, F=0.5311, p=0.4709, F=2.571, p=0.1176, respectively; Fig.2I).

4.2.4 Stress-related readouts and parameters of neuroinflammation

Overall there was no significant effect of stress, treatment or on interaction in the vehicle- or FS-treated animals for diet intake (p>0.05, two-way ANOVA; data not shown). However, there was a significant effect of stress on diet intake between week 1 and 3 (F=7.551, p=0.0106), but not at other time points (data not shown). Two-way ANOVA revealed that the by the end of stress procedure, on week three, body weight had been significantly affected by stress (F­­1,36=16.18, p<0.01, Fig. 3A), but not by treatment (F­­1,36=0.23, p=0.63) and there was no interaction (F­1,36=0.59, p=0.45). Post-hoc testing revealed a significant decrease in body weight in the US-vehicle treated group compared to the control vehicle group (p<0.01, Šídák's test), but not in the US-FS mice compared to the control FS-treated animals (p>0.05, Šídák's test).

Cortisol blood level was significantly affected by the stress factor (F1,35=7.718, p=0.0112, two-way ANOVA. Fig.3B). No effect of the FS treatment or a treatment x stress was found (F1,35=0.96, p=0.33 and F1,35=0.007, p=0.93, respectively). The expression of GSK-3β was no significantly affected in the PFC or in the hippocampus (stress factor: F­1,36=2.19, p=0.15; treatment factor: F­1,36=0.70, p=0.41; treatment x stress interaction F­1,36=2.64, p=0.11, Fig. 3C).

The expression of Il-1β in the PFC was significantly affected by the treatment (F=7.278, p=0.0106), stress (F=18.4, p=0.0001) and there was a treatment x stress interaction (F1,36= F=4.365, p=0.0438, two-way ANOVA. Fig. 3D). In the US-vehicle treated group, Il-1β expression was significantly increased compared to both control vehicle group (p=0.0004, Tukey's test) and US-FS-treated animals (p=0.009, Tukey's test). In the hippocampus, stress significantly increased Il-1β expression (F­1,36=6.63, p=0.0144, two-way ANOVA. Fig. 3D). No significant effects of treatment (F­1,36=2.01, p=0.16) or a treatment x stress interaction (F­1,36=1.29, p=0.26) were observed for Il-1β. A significant increase of expression in the hippocampus was found in the US-vehicle treated group compared to the control vehicle mice (p=0.02, Šídák's test), but not in the US-FS group compared to the control FS mice (p=0.5, Šídák's test). The expression of TNF in both the PFC and hippocampus, was significantly affected by the stress (PFC: F­1,36= F=8.697, p=0.0056; hippocampus: F1,36=5.59, p=0.02, two-way ANOVA. Fig. 3G, H), but not by treatment (PFC: F­1,36=1.58, p=0.22; hippocampus: F1,36=0.007, p=0.93), nor was there a treatment x stress interaction (PFC: F­1,36=1.35, p=0.25; hippocampus: F1,36=1.26, p=0.27). In both brain structures, significantly increased TNF expression was observed in the US-vehicle group compared to the control vehicle animals (PFC: p=0.01; hippocampus: p=0.04, Šídák's test). No significant differences were found between control FS and US-FS animals (PFC: p=0.38; hippocampus: p=0.62, Šídák's test). No other significant group differences were found using two-way ANOVA and post-hoc analysis of the TNF results.

## 4.3 Metabolome changes in brain and periphery

To understand the effect of stress and the omega-3 treatment on the metabolome, principal component analysis was conducted to inspect the metabolome data in the four groups of animals. No unsupervised group differences were identified (Fig. S1). However, subsequent machine learning random forest analysis was used to identify key metabolites in the plasma, liver, and brain that were altered by US and/or FS. A summary of the classification accuracies (ROC AUC) is shown in Table S2 (see Supplementary file), and a heatmap (z-scaled metabolite relative spectral intensity) of the most important metabolites identified across the plasma, liver, and brain is shown in Fig. S2 for the different groups.

### 4.3.1 Plasma metabolome

In the plasma, a random forest approach with a 5-fold cross-validation did not discriminate between stressed and non-stressed mice (AUC < 0.5, Fig. 4A), thus no further investigation into metabolite differences were performed in this dimension. Conversely, a significant difference was identified in the plasma between mice treated with Omega 3 and vehicle (AUC 0.58, Fig. 4B). This difference was driven by a significant decrease in the -CH_3_ signal from lipoproteins in mice treated with the Omega 3 supplement compared to mice treated with the vehicle (Fig. 4C, 4D).

### 4.3.2 Liver metabolome

In the liver, a random forest analysis with 5-fold cross-validation classified stressed and not-stressed animals with a mean AUC of 0.56 (Fig. 5A). Univariate inspection of the most important variables driving this model showed that choline and 3-hydroxybutyric acid contributed most to model accuracy (Fig. 5B). 3-hydroxybutyric acid was significantly reduced by US (Fig. 5E), whereas choline was significantly increased by stress (Fig. 5F). Animals treated with Omega 3 were distinguished from animals treated with vehicle with a ROC AUC of 0.76 (Fig. 5C). Glutamine, glucose, acetate, and taurine were the metabolites contributing most to the random forest model (Fig. 5D). Specifically, glucose and acetate were significantly reduced in the liver in treated mice compared to untreated mice (Fig. 5H and 5I respectively), whereas taurine was increased (Fig. 5J). A significant US x FS interaction occurred for liver glutamine levels, whereby stressed mice treated with Omega 3 had significantly higher levels compared to all other animals (Fig. 5G).

### 4.3.3 Brain metabolome

In the brain, mice subjected to US could be distinguished from non-stressed animals with a mean ROC AUC of 0.66 (Fig. 6A). Serine and glucose resonances contributed most to the accuracy of the random forest models generated (Fig. 6B). Serine was significantly increased in stressed animals compared to non-stressed animals (Fig. 6E) while brain glucose was decreased (Fig. 6F). The brain metabolome composition was also significantly altered by the omega 3 supplementation compared to vehicle (ROC AUC 0.76, Fig. 6C). The sphingomyelin resonances were the most important contributors to the model accuracy. Univariate analysis of sphingomyelin revealed the presence of a significant interaction; the concentration was increased in the stressed treated animals compared to all other groups (Fig. 6G).

4.3.4 Behavior-metabolome correlation analysis

Lastly, we investigated how key brain, liver, and plasma metabolites correlated with measures of anxiety, exploratory, and depressive-like behavior (Fig. 7). Brain glucose showed a significant positive correlation with exits within the dark/light box (reduced anxious behavior), whereas -the CH_3_ signal from lipoproteins in the blood correlated negatively with grid crossings in the open field and positively with freezing time in this test. In the liver, acetate and choline exhibited a negative correlation with time spent in the centre of the open field, and were therefore positively correlated with anxiety-like behaviors. Liver choline was also negatively correlated with sucrose preference and the number of exits in the dark/light box. Taurine was negatively correlated with rearing in the novel cage test, and liver glucose was negatively correlated with the number of crossings in the open field (Fig. 7).

## 5. Discussion

## Our study underscores the potential of omega-3 fatty acids to ameliorate depressive-like signs in mice and anxiety in conditions induced by emotional stress. The substantial neurobiological and metabolic alterations observed point to a complex interplay between omega-3 fatty acids and the stress-related pathophysiological processes. In particular, we noted pronounced effects of the stress and treatment interactions, primarily on our chosen markers of neuroinflammation, and illustrating a complex relationship between behavior and the metabolome. Importantly, the US placebo-treated group exhibited reduced sucrose preference—a hallmark of anhedonia and a key symptom of depression—which was effectively mitigated by omega-3 treatment in stressed mice. It is of note that the single housing, employed in these adolescent mice, may have also had an additive effect on the US stress, suggesting that this combination of factors may play a critical role in the modulation of stress responses and treatment efficacy.

## Our results are in agreement with previous findings in adult mouse models, where depression-like features responsive to antidepressant treatments have been noted (Strekalova et al., 2018; Gorlova et al., 2019; de Munter et al., 2021). By applying the US paradigm in juvenile mice, our study suggests the induction of an MDD-like syndrome, accompanied by heightened anxiety-like behavior. Additionally, the anti-anxiety effects of the omega-3 containing food supplement in non-stressed mice underscore the potential of omega-3 faty acids as a preventive measure as well as a therapeutic intervention. The behavioral changes we observed, such as improvements in anxiety-like behavior in the dark-light and open field tests, along with the normalisation of locomotor activity, grooming scores, and body weight, mirror depressive syndromes reported in both humans and adult animal models (Chaudhury et al., 2015; Strekalova et al., 2022). These behavioral modifications were accompanied by metabolic shifts in the brain, serum, and liver, highlighting the broad whole-body impact of omega-3 on metabolic pathways. The expression of pro-inflammatory cytokines, notably IL-1β and TNF, was significantly moderated by omega-3 supplementation, aligning with previous reports (So et al., 2021; Colasanto et al., 2020; Ferencova et al., 2022; Lamers et al., 2019), although the stress-induced rise in blood corticosterone levels remained unaltered. This discrepancy suggests that while omega-3 can mitigate some inflammatory responses, it may not affect all stress-related hormonal pathways.

## This study has been the first to explore the impact of omega-3 fatty acids on the brain metabolome so extensively, revealing distinct effects of stress and the food supplement. In essence, omega-3 treatment did not counteract the stress-induced changes in the metabolic profile of stressed mice. Instead, it influenced several parameters that stress had not altered, which independently appear to exert a protective effect. This is exemplified by the notable increase in sphingomyelin concentrations in the brain following omega-3 administration, a change that the US exposure did not affect. On the other hand, exposure to stress led to increased levels of serine and decreased levels of glucose, effects that omega-3 supplementation did not modify. The elevated concentrations sphingomyelin in the brain, induced by the omega-3 fatty acids, may have protective and behavior-normalising effects in rodents under chronic stress. This might be attributed to the role of omega-3 fatty acids in maintaining neuronal membrane fluidity, but it is more likely to be the way it influences the enzymes involved in sphingomyelin metabolism (Peña et al., 1997) that generate ceramide and sphingosine-1-phoaspahte. Previous studies have shown that omega-3 fatty acids have ability to downregulate sphingomyelinases, which break down sphingomyelin, thus mitigating inflammation (Opreanu et al., 2010; Kelly et al., 2011). Moreover, these effects can also underpin the previously demonstrated impact of omega-3 fatty acids in influencing neurodevelopment in a model of maternal immune activation (MIA) (Labrousse et al., 2018). In this LPS-induced MIA paradigm, omega-3 deficiency altered the fatty acid composition of the foetal and adult offspring brain, and exacerbated cytokine production and resulted in spatial memory deficits in the adult offspring (Labrousse et al., 2018).

## The reason for the effects of omega-3 on the metabolome in the periphery are less clear. Others have shown that omega-3 fatty acids (DHA in particular) prevented DNA damage and oxidative stress in liver cells, and significantly reduced liver injury in mice subject to an experimental model of liver injury. These hepatoprotective effects were associated with a decrease in the hepatic formation of omega-6-derived eicosanoids and a concomitant increase in the generation of protective DHA-derived lipid mediators by macrophages (González-Périz et al., 2006). Here, however, the omega-3 fatty acids were found to influence amino acid metabolism in the liver and to affect the synthesis of taurine, another important amino acid in the liver. Taurine is involved in bile acid conjugation, osmoregulation, and antioxidation. Omega-3 fatty acids are known to affect N-acyl taurines (NATs), which are bioactive lipids with emerging roles in glucose homeostasis and lipid metabolism (Trammell et al., 2023).

## Omega-3 fatty acids are also known to improve insulin sensitivity. This can lead to more efficient uptake and utilization of glucose by cells, thereby reducing the glucose levels in the liver as observed in our study. Additionally, omega-3 fatty acids might influence gluconeogenesis, the process of producing glucose from non-carbohydrate sources, leading to a decrease in glucose production in the liver. Acetate levels might also be reduced in the liver owing to the altered carbohydrate and fat metabolism influenced by the omega-3s. In light of our findings and the intriguing correlations observed between omega-3 supplementation and specific metabolomic profiles, further studies are now underway to explore the functional impacts of individual metabolites, such as taurine and acetate to dissect the mechanistic pathways through which omega-3 fatty acids influence health outcomes.

## Clinical research has linked adolescent depression with excessive production of pro-inflammatory cytokines (Colasanto et al., 2020; Ferencova et al., 2022; Lamers et al., 2019). Our findings concerning the effect of Omega-3 on the expression of IL-1β and TNF further contribute to this narrative. For instance, a study on adolescent patients with the first-episode of depression highlighted a correlation between pro-inflammatory TNF- and IL-6 signaling and increased anti-inflammatory activity mediated by IL-10 and IL-4 (Ferencova et al., 2022). Our results align with these findings, suggesting a broader applicability of omega-3 fatty acids in moderating inflammation-associated depressive symptoms in both humans and in our animal models.

## Conclusions

In conclusion, our study not only reveals the efficacy of omega-3 in countering stress-induced depressive and anxiety-like behaviors in adolescent mice, but also contributes significantly to our understanding of the role of omega-3 on neuroinflammatory and metabolic pathways, resonating with observations in human adolescent depression. These findings position omega-3 fatty acids as a promising avenue for future research and potential therapeutic interventions in stress-related mood disorders, advocating for a broader integration of nutritional strategies in mental health management..

**7. Acknowledgements**

This study was supported by Research Russia-Switzerland Grant-2020 (all experimental work was carried out by January 2022) to SW and TS, RF-0520-2019-0013 and PhytoAPP EU framework (2021-2025, to TS, and DA). The PhytoAPP project has received funding from the European Union’s HORIZON 2020 research and innovation program under the Marie Skłodowvska-Curie grant agreement 101007642. This research was funded by the Ministry of Science and Higher Education of the Russian Federation (Grant Number: 075-15-2022-310). We thank Ms Anastasia Ochertova for valuable technical contribution to this work. The sponsors had no role in study design; in the collection, analysis and interpretation of data; in the writing of the report; and in the decision to submit the article for publication.

**8. Author contributions**

Co-author contributions to the paper was following: TS, SW, DA – conceptualization; TS, DA, SM - Data curation; AG, ES, AL- Formal analysis; TS, SW, SM - Funding acquisition; AG, ID, DR, ESh, ES, AL - Investigation; TS, DA - Methodology; SM, TS, SW, SW - Project administration; SM, TS, DA, AL - Resources; DA -Software; TS, SW, SM, DA - Supervision; ESh, AG, ES -Validation; DA, AG, ES, AL -Visualization; TS, DA, AG, DA -Writing, original draft; SM, SW, DA, AL - Writing - review & editing.

**9. References**

Amminger, G.P., Rice, S., Davey, C.G., Quinn, A.L., Hermens, D.F., Zmicerevska, N., Nichles, A., Hickie, I., Incerti, L., Weller, A., Joseph, S., Hilton, Z., Pugh, C., Rayner, M., Reid, N., Ratheesh, A., Yung, A.R., Yuen, H.P., Mackinnon, A., Hetrick, S., Parker, A., Street, R., Berger, M., Berk, M., McGorry, P., Lin, A., 2024. The Addition of Fish Oil to Cognitive Behavioral Case Management for Youth Depression: A Randomized, Double-Blind, Placebo-Controlled, Multicenter Clinical Trial. Biological psychiatry 95, 426–433. <https://doi.org/10.1016/j.biopsych.2023.06.015>

Baune, B.T., Stuart, M., Gilmour, A., Wersching, H., Heindel, W., Arolt, V., Berger, K., 2012. The relationship between subtypes of depression and cardiovascular disease: a systematic review of biological models. Transl Psychiatry 2, e92. https://doi.org/10.1038/tp.2012.18

Bridge, J.A., Iyengar, S., Salary, C.B., Barbe, R.P., Birmaher, B., Pincus, H.A., Ren, L., Brent, D.A., 2007. Clinical Response and Risk for Reported Suicidal Ideation and Suicide Attempts in Pediatric Antidepressant Treatment. JAMA 297, 1683. https://doi.org/10.1001/jama.297.15.1683

Chang, J.P.-C., Su, K.-P., 2020. Nutritional Neuroscience as Mainstream of Psychiatry: The Evidence- Based Treatment Guidelines for Using Omega-3 Fatty Acids as a New Treatment for Psychiatric Disorders in Children and Adolescents. Clinical Psychopharmacology and Neuroscience 18, 469–483. <https://doi.org/10.9758/cpn.2020.18.4.469>

Chahboune, H., Ment, L.R., Stewart, W.B., Ma, X., Rothman, D.L., Hyder, F., 2007. Neurodevelopment of C57B/L6 mouse brain assessed by in vivo diffusion tensor imaging. NMR in biomedicine 20, 375–382. https://doi.org/10.1002/nbm.1130

Chaudhury, D., Liu, H., Han, M.-H., 2015. Neuronal correlates of depression. Cellular and Molecular Life Sciences 72, 4825–4848. https://doi.org/10.1007/s00018-015-2044-6

Cipriani, A., Zhou, X., Del Giovane, C., Hetrick, S.E., Qin, B., Whittington, C., Coghill, D., Zhang, Y., Hazell, P., Leucht, S., Cuijpers, P., Pu, J., Cohen, D., Ravindran, A. V, Liu, Y., Michael, K.D., Yang, L., Liu, L., Xie, P., 2016. Comparative efficacy and tolerability of antidepressants for major depressive disorder in children and adolescents: a network meta-analysis. The Lancet 388, 881–890. https://doi.org/10.1016/S0140-6736(16)30385-3

Colasanto, M., Madigan, S., Korczak, D.J., 2020. Depression and inflammation among children and adolescents: A meta-analysis. J Affect Disord 277, 940–948. https://doi.org/10.1016/j.jad.2020.09.025

Constantini, F., D’Amato, F.R., 2006. Ultrasonic vocalizations in mice and rats: social contexts and functions. Dong Wu Xue Bao 52, 619–633.

Costa-Nunes, J., Zubareva, O., Araújo-Correia, M., Valença, A., Schroeter, C.A., Pawluski, J.L., Vignisse, J., Steinbusch, H., Hermes, D., Phillipines, M., Steinbusch, H.M.W., Strekalova, T., 2014. Altered emotionality, hippocampus-dependent performance and expression of NMDA receptor subunit mRNAs in chronically stressed mice. Stress 17, 108–116. <https://doi.org/10.3109/10253890.2013.872619>

Costa-Nunes, J.P., Gorlova, A., Pavlov, D., Cespuglio, R., Gorovaya, A., Proshin, A., Umriukhin, A., Ponomarev, E.D., Kalueff, A.V., Strekalova, T., Schroeter, C.A., 2020. Ultrasound stress compromises the correlates of emotional-like states and brain AMPAR expression in mice: effects of antioxidant and anti-inflammatory herbal treatment. Stress (Amsterdam, Netherlands) 23, 481–495. <https://doi.org/10.1080/10253890.2019.1709435>

Couch, Y., Anthony, D.C., Dolgov, O., Revischin, A., Festoff, B., Santos, A.I., Steinbusch, H.W., Strekalova, T., 2013. Microglial activation, increased TNF and SERT expression in the prefrontal cortex define stress-altered behavior in mice susceptible to anhedonia. Brain Behav Immun 29, 136–146. https://doi.org/10.1016/j.bbi.2012.12.017

Cui, G., Qing, Y., Hu, X., Wang, P., Sun, L., Yang, X., Jiang, J., Zhang, J., Wang, H., Feng, L., Shen, Y., Qin, S., Wan, C., 2020. Serum Metabolomic Profiling Based on Fourier Transform-Ion Cyclotron Resonance-Mass Spectrometry: Do the Dysfunctions of Metabolic Pathways Reveal a Universal Risk of Oxidative Stress in Schizophrenia? Antioxid Redox Signal 33, 679–688. https://doi.org/10.1089/ars.2020.8141

de Graaf, R.A., Chowdhury, G.M.I., Behar, K.L., 2014. Quantification of high-resolution ^1^H-[^13^C] NMR spectra from rat brain extracts. Anal Chem 86, 5032–8. <https://doi.org/10.1021/ac5006926>

de Munter, J., Pavlov, D., Gorlova, A., Sicker, M., Proshin, A., Kalueff, A.V., Svistunov, A., Kiselev, D., Nedorubov, A., Morozov, S., Umriukhin, A., Lesch, K.P., Strekalova, T., Schroeter, C.A., 2021. Increased Oxidative Stress in the Prefrontal Cortex as a Shared Feature of Depressive- and PTSD-Like Syndromes: Effects of a Standardized Herbal Antioxidant. Frontiers in nutrition 8, 661455. https://doi.org/10.3389/fnut.2021.661455

Ferencova, N., Visnovcova, Z., Ondrejka, I., Funakova, D., Hrtanek, I., Kelcikova, S., Tonhajzerova, I., 2022. Evaluation of Inflammatory Response System (IRS) and Compensatory Immune Response System (CIRS) in Adolescent Major Depression. J Inflamm Res Volume 15, 5959–5976. <https://doi.org/10.2147/JIR.S387588>

González-Périz, A., Planagumà, A., Gronert, K., Miquel, R., López-Parra, M., Titos, E., Horrillo, R., Ferré, N., Deulofeu, R., Arroyo, V., Rodés, J., Clària, J., 2006. Docosahexaenoic acid (DHA) blunts liver injury by conversion to protective lipid mediators: protectin D1 and 17S-hydroxy-DHA. FASEB journal: official publication of the Federation of American Societies for Experimental Biology 20, 2537–2539. <https://doi.org/10.1096/fj.06-6250fje>

Gorlova, A., Pavlov, D., Anthony, D.C., Ponomarev, E.D., Sambon, M., Proshin, A., Shafarevich, I., Babaevskaya, D., Lesсh, K.P., Bettendorff, L., Strekalova, T., 2019. Thiamine and benfotiamine counteract ultrasound-induced aggression, normalize AMPA receptor expression and plasticity markers, and reduce oxidative stress in mice. Neuropharmacology 156, 107543. https://doi.org/10.1016/j.neuropharm.2019.02.025

Gorlova, A., Svirin, E., Pavlov, D., Cespuglio, R., Proshin, A., Schroeter, C.A., Lesch, K.-P., Strekalova, T., 2023. Understanding the Role of Oxidative Stress, Neuroinflammation and Abnormal Myelination in Excessive Aggression Associated with Depression: Recent Input from Mechanistic Studies. Int J Mol Sci 24, 915. https://doi.org/10.3390/ijms24020915

Govindaraju, V., Young, K., Maudsley, A.A., 2000. Proton NMR chemical shifts and coupling constants for brain metabolites. NMR Biomed 13, 129–53. https://doi.org/10.1002/1099-1492(200005)13:3<129::aid-nbm619>3.0.co;2-v

Häberling, I., Berger, G., Schmeck, K., Held, U., Walitza, S., 2019. Omega-3 Fatty Acids as a Treatment for Pediatric Depression. A Phase III, 36 Weeks, Multi-Center, Double-Blind, Placebo-Controlled Randomized Superiority Study. Frontiers in psychiatry 10, 863. <https://doi.org/10.3389/fpsyt.2019.00863>

Hankin, B.L., Young, J.F., Abela, J.R.Z., Smolen, A., Jenness, J.L., Gulley, L.D., Technow, J.R., Gottlieb, A.B., Cohen, J.R., Oppenheimer, C.W., 2015. Depression from childhood into late adolescence: Influence of gender, development, genetic susceptibility, and peer stress. J Abnorm Psychol 124, 803–816. <https://doi.org/10.1037/abn0000089>

Hueston, C.M., Cryan, J.F., Nolan, Y.M., 2017. Stress and adolescent hippocampal neurogenesis: diet and exercise as cognitive modulators. Translational psychiatry 7, e1081. https://doi.org/10.1038/tp.2017.48

Janiaud, P., Cornu, C., Lajoinie, A., Djemli, A., Cucherat, M., Kassai, B., 2017. Is the perceived vehicle effect comparable between adults and children? A meta-regression analysis. Pediatr Res 81, 11–17. https://doi.org/10.1038/pr.2016.181

Kelly, L., Grehan, B., Chiesa, A. Della, O’Mara, S.M., Downer, E., Sahyoun, G., Massey, K.A., Nicolaou, A., Lynch, M.A., 2011. The polyunsaturated fatty acids, EPA and DPA exert a protective effect in the hippocampus of the aged rat. Neurobiol Aging 32, 2318.e1-2318.e15. https://doi.org/10.1016/j.neurobiolaging.2010.04.001

Kronenberg, S., Apter, A., Brent, D., Schirman, S., Melhem, N., Pick, N., Gothelf, D., Carmel, M., Frisch, A., Weizman, A., 2007. Serotonin Transporter Polymorphism (5-HTTLPR) and Citalopram Effectiveness and Side Effects in Children with Depression and/or Anxiety Disorders. J Child Adolesc Psychopharmacol 17, 741–750. https://doi.org/10.1089/cap.2006.0144

Labrousse, V.F., Leyrolle, Q., Amadieu, C., Aubert, A., Sere, A., Coutureau, E., Grégoire, S., Bretillon, L., Pallet, V., Gressens, P., Joffre, C., Nadjar, A., Layé, S., 2018. Dietary omega-3 deficiency exacerbates inflammation and reveals spatial memory deficits in mice exposed to lipopolysaccharide during gestation. Brain Behav Immun 73, 427–440. https://doi.org/10.1016/j.bbi.2018.06.004

Lamers, F., Milaneschi, Y., Smit, J.H., Schoevers, R.A., Wittenberg, G., Penninx, B.W.J.H., 2019. Longitudinal Association Between Depression and Inflammatory Markers: Results From the Netherlands Study of Depression and Anxiety. Biol Psychiatry 85, 829–837. https://doi.org/10.1016/j.biopsych.2018.12.020

LeMoult, J., Humphreys, K.L., Tracy, A., Hoffmeister, J.-A., Ip, E., Gotlib, I.H., 2020. Meta-analysis: Exposure to Early Life Stress and Risk for Depression in Childhood and Adolescence. J Am Acad Child Adolesc Psychiatry 59, 842–855. https://doi.org/10.1016/j.jaac.2019.10.011

Li, K., Zhou, G., Xiao, Y., Gu, J., Chen, Q., Xie, S., Wu, J., 2022. Risk of Suicidal Behaviors and Antidepressant Exposure Among Children and Adolescents: A Meta-Analysis of Observational Studies. Front Psychiatry 13. <https://doi.org/10.3389/fpsyt.2022.880496>

Lin, P.Y., Huang, S.Y., Su, K.P., 2010. A meta-analytic review of polyunsaturated fatty acid compositions in patients with depression. Biological psychiatry 68, 140–147. https://doi.org/10.1016/j.biopsych.2010.03.018

Locher, C., Koechlin, H., Zion, S.R., Werner, C., Pine, D.S., Kirsch, I., Kessler, R.C., Kossowsky, J., 2017. Efficacy and Safety of Selective Serotonin Reuptake Inhibitors, Serotonin-Norepinephrine Reuptake Inhibitors, and Vehicle for Common Psychiatric Disorders Among Children and Adolescents. JAMA Psychiatry 74, 1011. https://doi.org/10.1001/jamapsychiatry.2017.2432

Lu, W., 2019. Adolescent Depression: National Trends, Risk Factors, and Healthcare Disparities. Am J Health Behav 43, 181–194. https://doi.org/10.5993/AJHB.43.1.15

Madigan, S., Racine, N., Vaillancourt, T., Korczak, D.J., Hewitt, J.M.A., Pador, P., Park, J.L., McArthur, B.A., Holy, C., Neville, R.D., 2023. Changes in Depression and Anxiety Among Children and Adolescents From Before to During the COVID-19 Pandemic. JAMA Pediatr 177, 567. https://doi.org/10.1001/jamapediatrics.2023.0846

Maggu, G., Verma, V., Chaudhury, S., Indla, V., 2023. Epidemic of Depression and Anxiety in child and adolescent population during COVID-19 pandemic: A systematic review and meta analysis of the prevalence of depression and anxiety. Indian J Psychiatry 65, 299. https://doi.org/10.4103/indianjpsychiatry.indianjpsychiatry_700_21

Malatynska, E., Steinbusch, H.W.M., Redkozubova, O., Bolkunov, A., Kubatiev, A., Yeritsyan, N.B., Vignisse, J., Bachurin, S., Strekalova, T., 2012. Anhedonic-like traits and lack of affective deficits in 18-month-old C57BL/6 mice: Implications for modeling elderly depression. Exp Gerontol 47, 552–64. https://doi.org/10.1016/j.exger.2012.04.010

Masi, G., 2022. Controversies in the Pharmacotherapy of Adolescent Depression. Curr Pharm Des 28, 1975–1984. https://doi.org/10.2174/1381612828666220526150153

Meister, R., Abbas, M., Antel, J., Peters, T., Pan, Y., Bingel, U., Nestoriuc, Y., Hebebrand, J., 2020. Vehicle response rates and potential modifiers in double-blind randomized controlled trials of second and newer generation antidepressants for major depressive disorder in children and adolescents: a systematic review and meta-regression analysis. Eur Child Adolesc Psychiatry 29, 253–273. https://doi.org/10.1007/s00787-018-1244-7

Morozova, A., Zubkov, E., Strekalova, T., Kekelidze, Z., Storozeva, Z., Schroeter, C.A., Bazhenova, N., Lesch, K.-P., Cline, B.H., Chekhonin, V., 2016. Ultrasound of alternating frequencies and variable emotional impact evokes depressive syndrome in mice and rats. Prog Neuropsychopharmacol Biol Psychiatry 68, 52–63. https://doi.org/10.1016/j.pnpbp.2016.03.003

Nakatake, Y., Furuie, H., Yamada, Misa, Kuniishi, H., Ukezono, M., Yoshizawa, K., Yamada, Mitsuhiko, 2020. The effects of emotional stress are not identical to those of physical stress in mouse model of social defeat stress. Neurosci Res 158, 56–63. https://doi.org/10.1016/j.neures.2019.10.008

Oliveira, J.M.D. de, Butini, L., Pauletto, P., Lehmkuhl, K.M., Stefani, C.M., Bolan, M., Guerra, E., Dick, B., De Luca Canto, G., Massignan, C., 2022. Mental health effects prevalence in children and adolescents during the COVID‐19 pandemic: A systematic review. Worldviews Evid Based Nurs 19, 130–137. https://doi.org/10.1111/wvn.12566

Opreanu, M., Lydic, T.A., Reid, G.E., McSorley, K.M., Esselman, W.J., Busik, J. V., 2010. Inhibition of Cytokine Signaling in Human Retinal Endothelial Cells through Downregulation of Sphingomyelinases by Docosahexaenoic Acid. Investigative Opthalmology & Visual Science 51, 3253. https://doi.org/10.1167/iovs.09-4731

Pavlov, D., Bettendorff, L., Gorlova, A., Olkhovik, A., Kalueff, A. V., Ponomarev, E.D., Inozemtsev, A., Chekhonin, V., Lesсh, K.P., Anthony, D.C., Strekalova, T., 2019. Neuroinflammation and aberrant hippocampal plasticity in a mouse model of emotional stress evoked by exposure to ultrasound of alternating frequencies. Prog Neuropsychopharmacol Biol Psychiatry 90, 104–116. https://doi.org/10.1016/j.pnpbp.2018.11.014

Pavlov, D., Gorlova, A., Haque, A., Cavalcante, C., Svirin, E., Burova, A., Grigorieva, E., Sheveleva, E., Malin, D., Efimochkina, S., Proshin, A., Umriukhin, A., Morozov, S., Strekalova, T., 2023. Maternal Chronic Ultrasound Stress Provokes Immune Activation and Behavioral Deficits in the Offspring: A Mouse Model of Neurodevelopmental Pathology. Int J Mol Sci 24, 11712. <https://doi.org/10.3390/ijms241411712>

Peña, L.A., Fuks, Z., Kolesnick, R., 1997. Stress-induced apoptosis and the sphingomyelin pathway. Biochemical pharmacology 53, 615–621. <https://doi.org/10.1016/s0006-2952(96)00834-9>

Probert, F., Gorlova, A., Deikin, A., Bettendorff, L., Veniaminova, E., Nedorubov, A., Chaprov, K.D., Ivanova, T.A., Anthony, D.C., Strekalova, T., 2022. In FUS[1-359]-tg mice O,S-dibenzoyl thiamine reduces muscle atrophy, decreases glycogen synthase kinase 3 beta, and normalizes the metabolome. Biomed Pharmacother 156, 113986. https://doi.org/10.1016/j.biopha.2022.113986

Pruneti, C., Guidotti, S., 2023. Need for Multidimensional and Multidisciplinary Management of Depressed Preadolescents and Adolescents: A Review of Randomized Controlled Trials on Oral Supplementations (Omega-3, Fish Oil, Vitamin D3). Nutrients 15, 2306. https://doi.org/10.3390/nu15102306

Racine, N., McArthur, B.A., Cooke, J.E., Eirich, R., Zhu, J., Madigan, S., 2021. Global Prevalence of Depressive and Anxiety Symptoms in Children and Adolescents During COVID-19. JAMA Pediatr 175, 1142. https://doi.org/10.1001/jamapediatrics.2021.2482

Rice, F., Riglin, L., Lomax, T., Souter, E., Potter, R., Smith, D.J., Thapar, A.K., Thapar, A., 2019. Adolescent and adult differences in major depression symptom profiles. J Affect Disord 243, 175–181. <https://doi.org/10.1016/j.jad.2018.09.015>

Sambon, M., Gorlova, A., Demelenne, A., Alhama-Riba, J., Coumans, B., Lakaye, B., Wins, P., Fillet, M., Anthony, D.C., Strekalova, T., Bettendorff, L., 2020. Dibenzoylthiamine Has Powerful Antioxidant and Anti-Inflammatory Properties in Cultured Cells and in Mouse Models of Stress and Neurodegeneration. Biomedicines 8, 361. <https://doi.org/10.3390/biomedicines8090361>

Semple, B.D., Blomgren, K., Gimlin, K., Ferriero, D.M., Noble-Haeusslein, L.J., 2013. Brain development in rodents and humans: Identifying benchmarks of maturation and vulnerability to injury across species. Progress in neurobiology 106-107, 1–16. https://doi.org/10.1016/j.pneurobio.2013.04.001

Sher, L., 2011. The role of brain-derived neurotrophic factor in the pathophysiology of adolescent suicidal behavior. Int J Adolesc Med Health 23. https://doi.org/10.1515/ijamh.2011.041

Shorey, S., Ng, E.D., Wong, C.H.J., 2022. Global prevalence of depression and elevated depressive symptoms among adolescents: A systematic review and meta‐analysis. British Journal of Clinical Psychology 61, 287–305. https://doi.org/10.1111/bjc.12333

So, J., Wu, D., Lichtenstein, A.H., Tai, A.K., Matthan, N.R., Maddipati, K.R., Lamon-Fava, S., 2021. EPA and DHA differentially modulate monocyte inflammatory response in subjects with chronic inflammation in part via plasma specialized pro-resolving lipid mediators: A randomized, double-blind, crossover study. Atherosclerosis 316, 90–98. https://doi.org/10.1016/J.ATHEROSCLEROSIS.2020.11.018

Soininen, P., Kangas, A.J., Würtz, P., Tukiainen, T., Tynkkynen, T., Laatikainen, R., Järvelin, M.-R., Kähönen, M., Lehtimäki, T., Viikari, J., Raitakari, O.T., Savolainen, M.J., Ala-Korpela, M., 2009. High-throughput serum NMR metabonomics for cost-effective holistic studies on systemic metabolism. Analyst 134, 1781. https://doi.org/10.1039/b910205a

Souza, L.C., Galvão, L.P., Paiva, H.S., Périco, C. de A.-M., Ventriglio, A., Torales, J., Castaldelli-Maia, J.M., Martins-da-Silva, A.S., 2023. Major depressive disorder as a risk factor for suicidal ideation for attendees of educational institutions: a meta-analysis and meta-regression. Revista Paulista de Pediatria 41. https://doi.org/10.1590/1984-0462/2023/41/2021344

Spielmans, G.I., Spence-Sing, T., Parry, P., 2020. Duty to Warn: Antidepressant Black Box Suicidality Warning Is Empirically Justified. Front Psychiatry 11. https://doi.org/10.3389/fpsyt.2020.00018

Strekalova, T., Bahzenova, N., Trofimov, A., Schmitt-Böhrer, A.G., Markova, N., Grigoriev, V., Zamoyski, V., Serkova, T., Redkozubova, O., Vinogradova, D., Umriukhin, A., Fisenko, V., Lillesaar, C., Shevtsova, E., Sokolov, V., Aksinenko, A., Lesch, K.P., Bachurin, S., 2018. Pro-neurogenic, memory-enhancing and anti-stress effects of DF302, a novel fluorine gamma-carboline derivative with multi-target mechanism of action. Mol Neurobiol 55, 335–349. https://doi.org/10.1007/s12035-017-0745-6

Strekalova, T., Couch, Y., Kholod, N., Boyks, M., Malin, D., Leprince, P., Steinbusch, H.M.W., 2011. Update in the methodology of the chronic stress paradigm: Internal control matters. Behavioral and Brain Functions 7, 9. https://doi.org/10.1186/1744-9081-7-9

Strekalova, T., Liu, Y., Kiselev, D., Khairuddin, S., Chiu, J.L.Y., Lam, J., Chan, Y.-S., Pavlov, D., Proshin, A., Lesch, K.-P., Anthony, D.C., Lim, L.W., 2022. Chronic mild stress paradigm as a rat model of depression: facts, artifacts, and future perspectives. Psychopharmacology (Berl) 239, 663–693. https://doi.org/10.1007/s00213-021-05982-w

Strekalova, T., Spanagel, R., Bartsch, D., Henn, F.A., Gass, P., 2004. Stress-induced anhedonia in mice is associated with deficits in forced swimming and exploration. Neuropsychopharmacology 29, 2007–2017. https://doi.org/10.1038/sj.npp.1300532

Strekalova, T., Spanagel, R., Dolgov, O., Bartsch, D., 2005. Stress-induced hyperlocomotion as a confounding factor in anxiety and depression models in mice. Behavioral Pharmacology 16, 171–180. https://doi.org/10.1097/00008877-200505000-00006

Strekalova, T., Steinbusch, H.W.M., 2010. Measuring behavior in mice with chronic stress depression paradigm. Prog Neuropsychopharmacol Biol Psychiatry 34, 348–61. https://doi.org/10.1016/j.pnpbp.2009.12.014

Strekalova, T., Svirin, E., Veniaminova, E., Kopeikina, E., Veremeyko, T., Yung, A.W.Y., Proshin, A., Walitza, S., Anthony, D.C., Lim, L.W., Lesch, K.-P., Ponomarev, E.D., 2021. ASD-like behaviors, a dysregulated inflammatory response and decreased expression of PLP1 characterize mice deficient for sialyltransferase ST3GAL5. Brain Behav Immun Health 16, 100306. https://doi.org/10.1016/j.bbih.2021.100306

Thakur, T., Mann, S.K., Malhi, N.K., Marwaha, R., 2023. The Role of Omega-3 Fatty Acids in the Treatment of Depression in Children and Adolescents: A Literature Review. Cureus. https://doi.org/10.7759/cureus.44584

Trammell, S.A.J., Gamon, L.F., Gotfryd, K., Michler, K.T., Alrehaili, B.D., Rix, I., Knop, F.K., Gourdon, P., Lee, Y.-K., Davies, M.J., Gillum, M.P., Grevengoed, T.J., 2023. Identification of bile acid-CoA:amino acid N-acyltransferase as the hepatic N-acyl taurine synthase for polyunsaturated fatty acids. J Lipid Res 64, 100361. https://doi.org/10.1016/j.jlr.2023.100361

Witteveen, A.B., Young, S.Y., Cuijpers, P., Ayuso-Mateos, J.L., Barbui, C., Bertolini, F., Cabello, M., Cadorin, C., Downes, N., Franzoi, D., Gasior, M., Gray, B., Melchior, M., van Ommeren, M., Palantza, C., Purgato, M., van der Waerden, J., Wang, S., Sijbrandij, M., 2023. COVID-19 and common mental health symptoms in the early phase of the pandemic: An umbrella review of the evidence. PLoS Med 20, e1004206. https://doi.org/10.1371/journal.pmed.1004206

Yang, L., Zhao, Y., Wang, Y., Liu, L., Zhang, X., Li, B., Cui, R., 2015. The Effects of Psychological Stress on Depression. Curr Neuropharmacol 13, 494–504. https://doi.org/10.2174/1570159X1304150831150507

Zuzarte, P., Duong, A., Figueira, M.L., Costa-Vitali, A., Scola, G., 2018. Current Therapeutic Approaches for Targeting Inflammation in Depression and Cardiovascular Disease. Curr Drug Metab 19, 674–687. https://doi.org/10.2174/1389200219666180305143501

Zwolińska, W., Dmitrzak-Węglarz, M., Słopień, A., 2023. Biomarkers in Child and Adolescent Depression. Child Psychiatry Hum Dev 54, 266–281. https://doi.org/10.1007/s10578-021-01246-y

**9. Figure Legends**

**Figure 1. Sequence of US experimental procedures and induction of the anhedonic state in mice. (A)** Schematic of the study flow and the use of in vivo and in vitro procedures. **(B)** Sucrose preference was significantly decreased in the US-stressed vehicle-treated group compared to both non-stressed vehicle-treated group and US-stressed FS-treated group, in a subgroup of mice (two-way ANOVA and Tukey test, *p<0.05). According to the criterion of 60% preference for sucrose solution over water (see the text), the group of stressed mice split into anhedonic and resilient to anhedonia subgroups. **(С)** Percent of anhedonic mice was significantly lower in FS-treated stressed group than in vehicle-treated stressed group (exact Fisher test, *p<0.05). **(E)** There was a significant effect of stress and treatment in the sucrose intake and **(F)** in water intake. Water intake was significantly higher in US-treated vehicle-fed group than in the respective control and stressed FS-treated mice (two-way ANOVA and Tukey test, *p<0.05 and # p<0.05). **(G)** No significant group differences were found in total intake suggesting that their drinking behavior was not compromised by US or FS treatment. Note ‘omega-3’= FS. Data is presented as mean ± SEM. Green bars: unstressed; blue bars: stressed.

**Figure 2. Changes in behavioral measures for emotionality and locomotion**. In the dark-light box **(A)** the latency of exit to lit box was significantly affected by stress **(B)** there was significant effect of treatment on the time spent in the lit box and **(C)** no significant effect by either factors nor stress x treatment interaction on the number of exits to the light box. In the open field test, **(D)** there was significant effect of treatment on the number of sectors, where US-vehicle group displayed significantly lower locomotion scores than US-FS groups, and control-FS and US-FS groups (*p<0.05, vs. US-vehicle group). **(E)** There was a significant effect of treatment and stress on the time spent in the center; Post-hoc test demonstrated significantly shorter time spent in the open field center in US-vehicle-treated group than in control vehicle-treated group in US-FS- animals and control-FS-treated mice (*p<0.05, vs. US-vehicle group); **(F)** the duration of freezing was significantly affected by treatment and stress, US-vehicle-treated group spent with freezing significantly longer time in than control vehicle-treated group (*p<0.05, vs. US-vehicle group), The duration of freezing was significantly shorter in control-FS group than in US-vehicle-treated group and US-FS group (#p<0.05, vs. control-FS group). **(G)** Treatment, stress and their interaction significant affected the frequency of grooming events that was significantly reduced in the US-vehicle-treated group than in the control vehicle-treated mice (*p<0.05, vs. US-vehicle group). **(H)** There was a significant effect of stress on the number of rears. **(I)**  In the novel cage, there were no significant effect of stress, treatment and their interaction were found in total number of rears. Two-way ANOVA, Šídák's test and Tukey's test. Note ‘omega-3’= FS. Data is presented as mean ± SEM. Green bars: unstressed; blue bars: stressed.

**Figure 3.** **Effects of FS on stress-related readouts, parameters of neuroinflammation and oxidative stress. (A)** By the end of stress, on week three, body weight was significantly affected by stress. US-vehicle treated mice, but not in but not US-FS treated animals displayed ssignificantly decreased body weight in comparison with respective control groups. **(B)** There was a significant effect of stress on blood cortisol level, but no significant group differences. **(C)** GSK-3β mRNA expression was not significantly changed nor in the PFC, nor in the hippocampus of experimental groups. **(D)** The expression of Il-1β in the PFC was significantly affected by treatment and treatment x stress interaction. In the hippocampus, there was significant stress effect on Il-1β expression that was significantly increased in the US-vehicle treated group compared to the control vehicle mice, but not in the US-FS group compared to the control FS mice. **(E)** Expression of TNF in both PFC and hippocampus was significantly affected by the stress and was significantly elevated in the US-vehicle group compared to the control vehicle animals, while there were no significant differences between control FS and US-FS groups. *p<0.05, two-way ANOVA with Šídák's test or Tukey's test. Animals n=10 in each group. Note ‘omega-3’= FS. Data is presented as mean ± SEM. Green bars: unstressed; blue bars: stressed.

**Figure 4.** Analysis of the plasma metabolome revealed no effect of US and a modest effect of FS driven by a change in -CH_3_ lipoprotein levels. Random forest-based machine learning analysis of plasma metabolites revealed no group differences according to US **(A)** and a significant difference according to FS **(B).** Inspection of the key metabolites driving this difference revealed the importance of -CH_3_ lipoprotein fatty acyl chains **(C),** which were significantly reduced in mice treated with FS compared to vehicle, independent of US (p < 0.01, **D**). N = 10 per group. Note ‘omega-3’= FS.

**Figure 5.** The liver metabolome was affected by both US and FS. Random forest-based machine learning analysis of liver metabolites revealed a modest effect of US **(A).** Group differences were driven by choline and 3-hydroxybutyric acid **(B).** FS strongly affected the liver metabolome **(C),** in particular the levels of glutamine, glucose, acetate, and taurine **(D).** Univariate analysis of key metabolites showed that 3-HB **(E)** was reduced in the liver by US, whereas free choline (F) was increased (both p < 0.05). Liver glutamine **(G)** was markedly increased by Omega-3 supplementation relative to vehicle (p < 0.001). There was also a significant interaction between FS and US, whereby the FS in animals subjected to US resulted in significantly increased glutamine levels relative to all other conditions. Glucose **(H)** and acetate **(I)** were decreased in FS-treated animals compared to those treated with vehicle (p < 0.05 and p < 0.01, respectively), whereas taurine was increased (p < 0.05). N = 10 per group. Note ‘omega-3’= FS.

**Figure 6.** The brain metabolome is distinctly affected by both US and FS. Random forest-based machine learning methods discriminate US from non-stressed animals with a ROC AUC of 0.66 (A) which is predominately driven by changes in brain serine and glucose levels (B). Similarly, multivariate analysis of the brain metabolome could distinguish mice treated with FS compared to vehicle with a ROC AUC of 0.76 **(C)** driven by changes in brain sphingomyelin (D). Specifically, US led to an increase in brain serine (**E**, p < 0.001) and a decrease in glucose (F, p < 0.01), while FS treatment increased brain sphingomyelin compared to vehicle (F, p < 0.0001). There was also a main effect of US on brain sphingomyelin levels, whereby stress increased sphingomyelin (p < 0.01), as well as a significant interaction (p < 0.05). Post-hoc analysis revealed that mice subjected to both US and FS had increased sphingomyelin levels relative to all other groups. N = 10 per group. Note ‘omega-3’= FS.

**Figure 7.** Correlation between key liver, plasma, or brain metabolites with behavioral outcomes. Novel cage rearing was negatively correlated with liver taurine levels (r = -0.40, p < 0.05). Exits in the dark light box (DLB) was negatively correlated with liver choline (r = -0.38, p < 0.05) and positively correlated with brain glucose (r = 0.41, p < 0.01). Sucrose preference was negatively correlated with liver choline (r = -0.40, p < 0.05). Grid crossings in the open field (OFT) was negatvely correlated with both liver glucose (r = -0.33, p < 0.05) and plasma -CH_3_ lipoprotein (r = -0.34, p < 0.05). Time in the centre of the OFT was negatively correlated with liver acetate (r = -0.39, p < 0.05) and liver choline (r = -0.54, p < 0.001), while time freezing in the OFT was positively correlated with plasma -CH_3_ lipoprotein (r = 0.44, p < 0.01).

**Figure S1.** Principal component analysis of plasma **(A),** liver **(B),** and brain **(C)** reveals no unsupervised group differences.

**Figure S2.**. Summary heatmap of key metabolites in the brain, liver, and plasma of mice exposed to vehicle/no stress, omega 3/no stress, vehicle/stress, or omega 3/stress. Scale bar shows z-scaled metabolite relative spectral intensity. N = 10 per group. Note ‘omega-3’= FS.


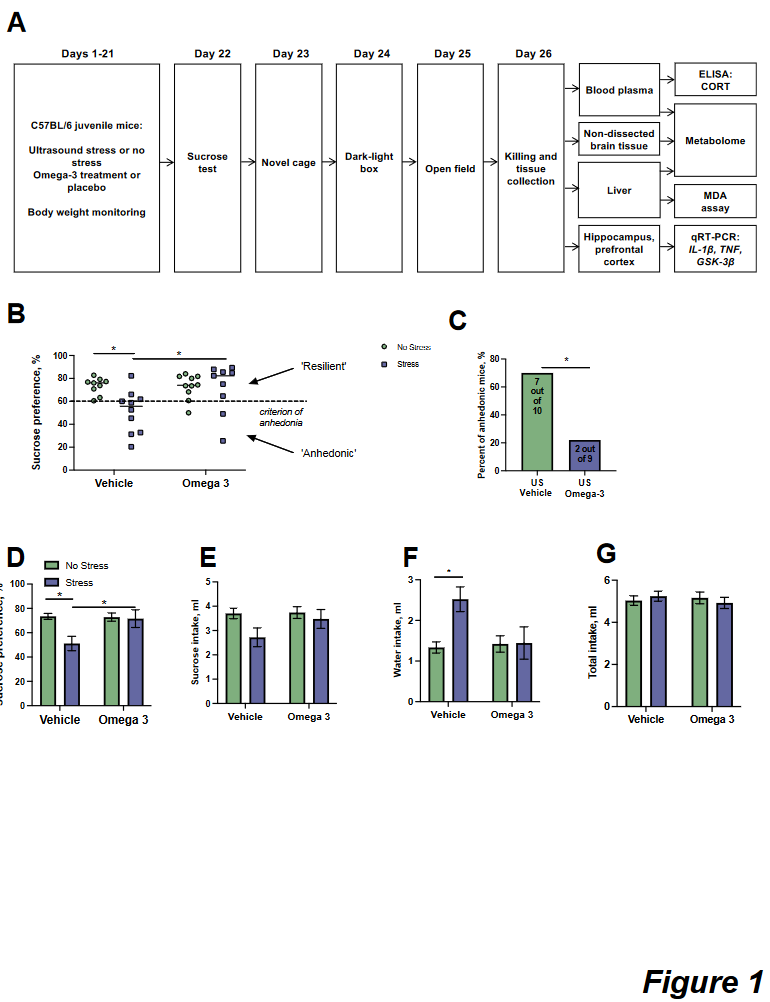

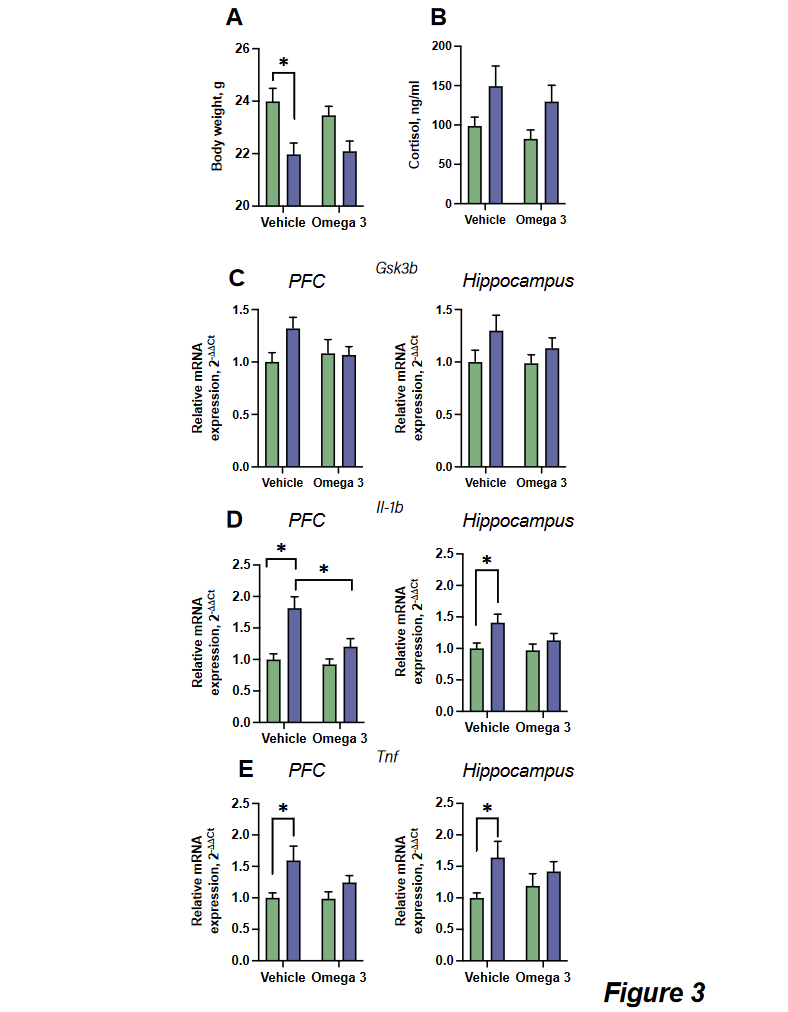


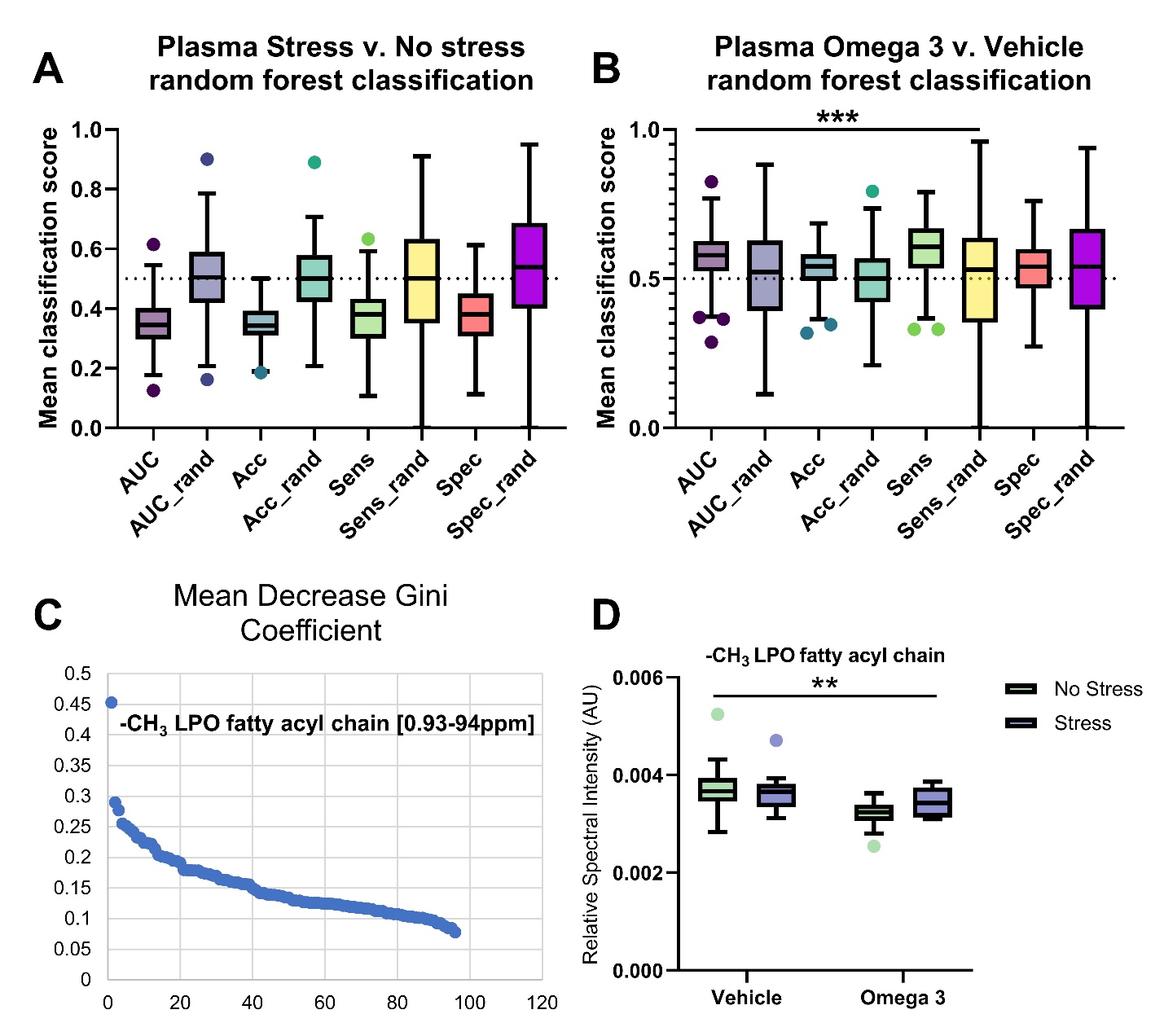


**Figure 4**


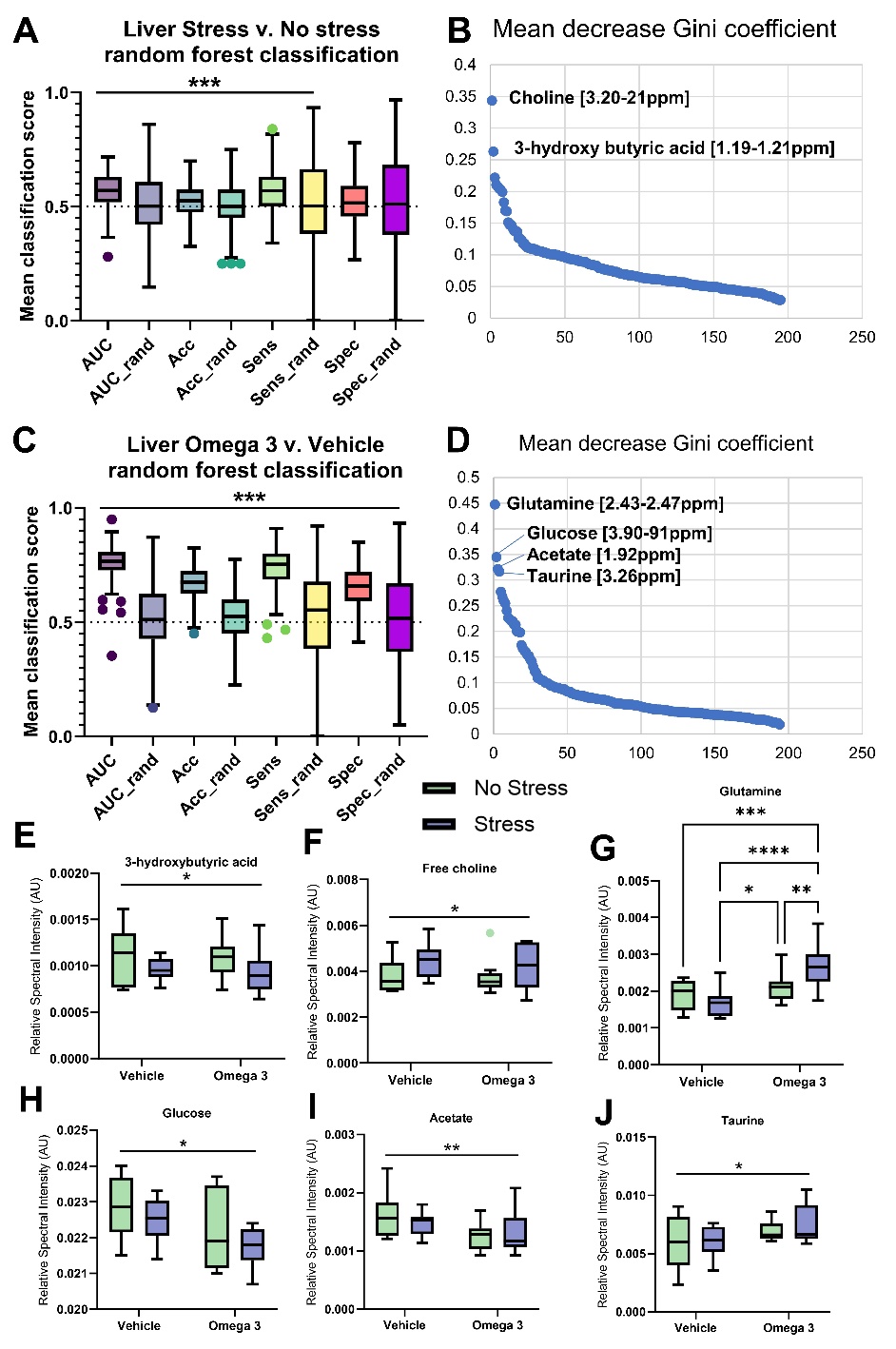


**Figure 5**


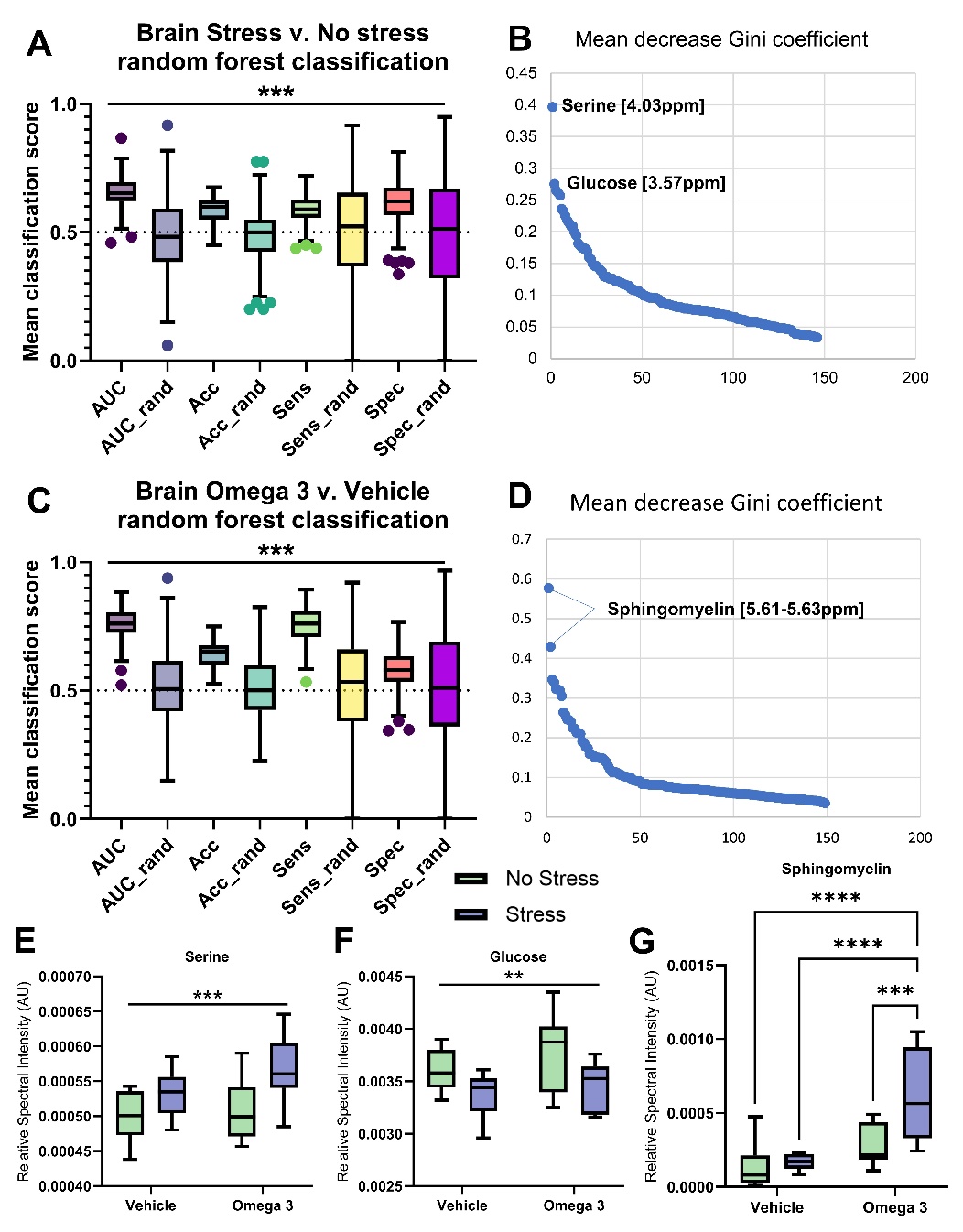


**Figure 6**


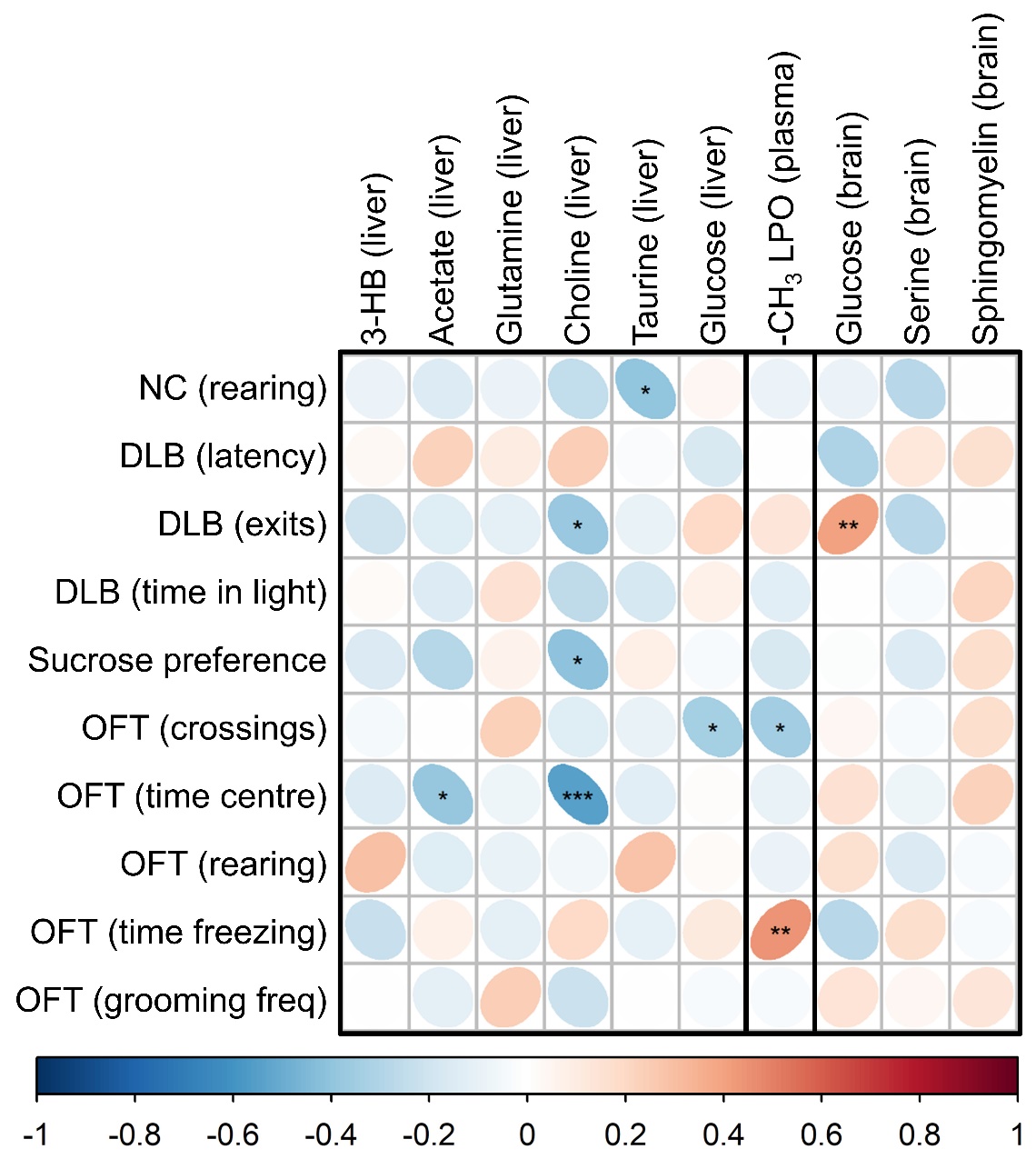


**Figure 7**


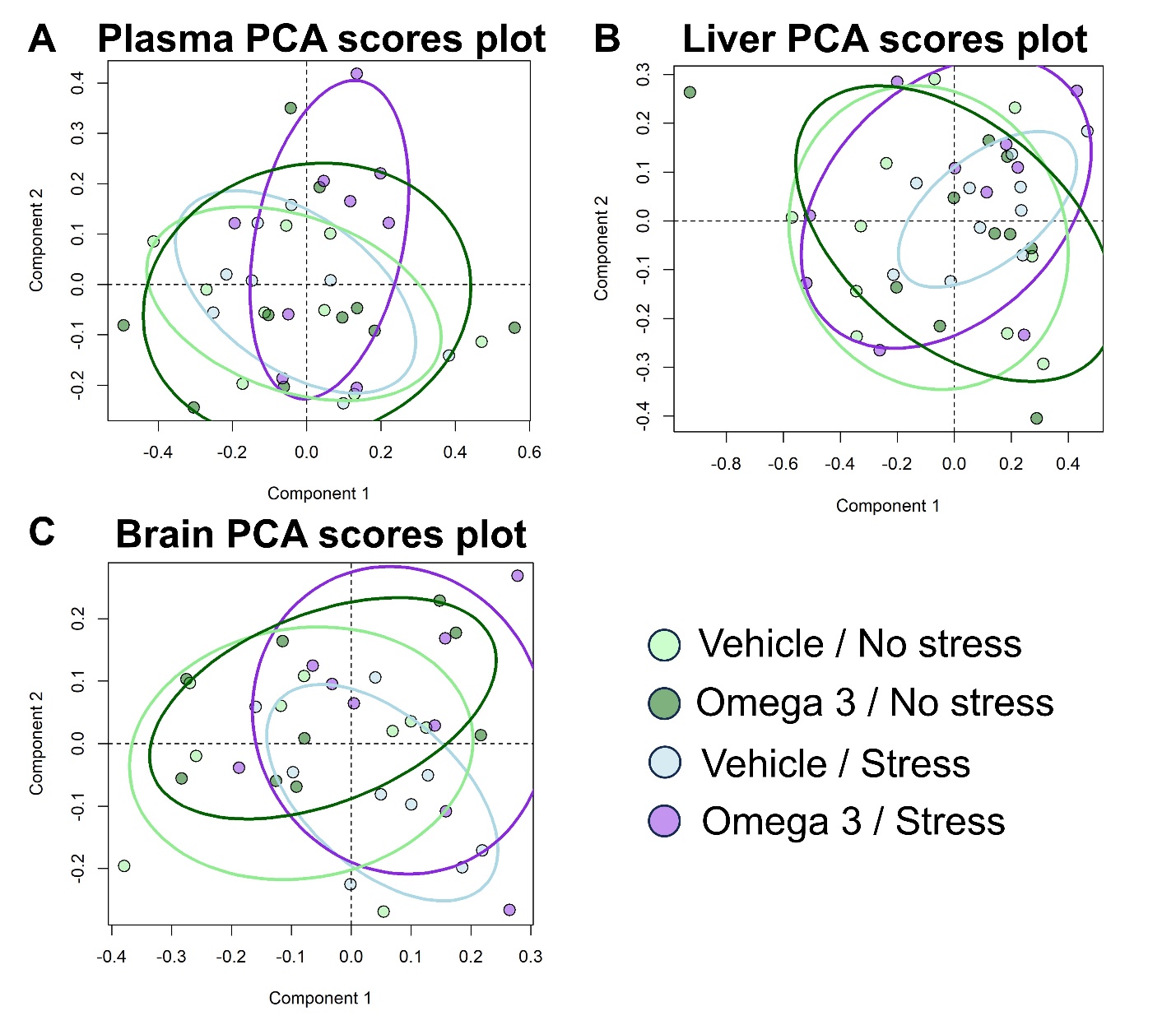


**Figure S1**


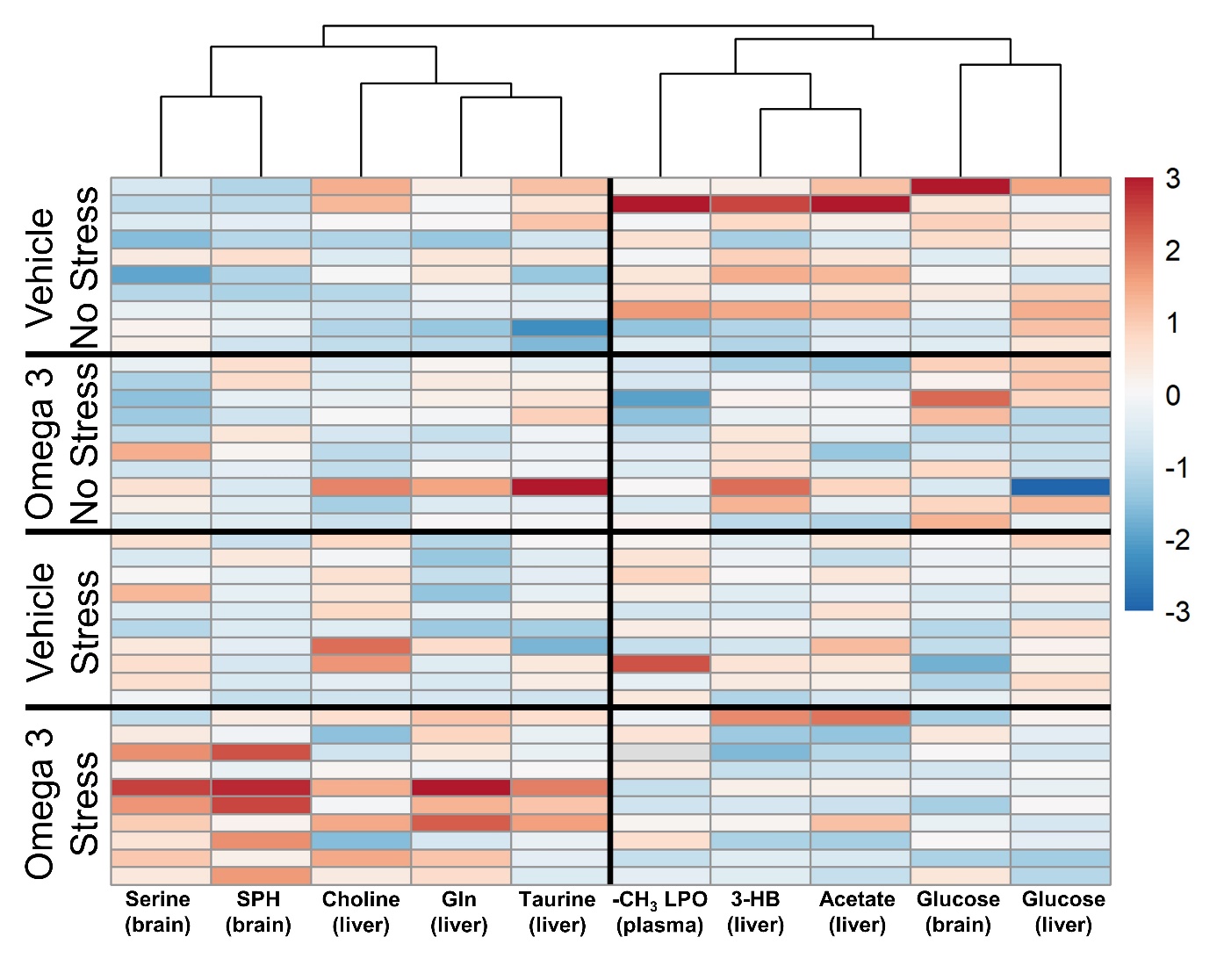


**Figure S2**
